# Alternative mRNA fates identified in microRNA-associated transcriptome analysis

**DOI:** 10.1186/1471-2164-13-561

**Published:** 2012-10-19

**Authors:** Adam P Carroll, Nham Tran, Paul A Tooney, Murray J Cairns

**Affiliations:** 1 School of Biomedical Sciences and Pharmacy, Faculty of Health and Hunter Medical Research Institute, University of Newcastle, Callaghan, NSW, Australia; 2 Schizophrenia Research Institute, Darlinghurst, NSW, Australia; 3 School Medical and Molecular Biosciences. Faculty of Science, University of Technology, Sydney, NSW, Australia; 4 Centre for Health Technologies, Faculty of Engineering and Information Technology, University of Technology, Sydney, Australia

**Keywords:** miRNA, miR-181b, E2F1, Target identification, Gene expression

## Abstract

**Background:**

MicroRNA (miRNA) are small non-coding RNA molecules which function as nucleic acid-based specificity factors in the universal RNA binding complex known as the RNA induced silencing complex (RISC). In the canonical gene-silencing pathway, these activated RISC particles are associated with RNA decay and gene suppression, however, there is evidence to suggest that in some circumstances they may also stabilise their target RNA and even enhance translation. To further explore the role of miRNA in this context, we performed a genome-wide expression analysis to investigate the molecular consequences of bidirectional modulation of the disease-associated miRNAs miR-181b and miR-107 in multiple human cell lines.

**Results:**

This data was subjected to pathways analysis and correlated against miRNA targets predicted through seed region homology. This revealed a large number of both conserved and non-conserved miRNA target genes, a selection of which were functionally validated through reporter gene assays. Contrary to expectation we also identified a significant proportion of predicted target genes with both conserved and non-conserved recognition elements that were positively correlated with the modulated miRNA. Finally, a large proportion of miR-181b associated genes devoid of the corresponding miRNA recognition element, were enriched with binding motifs for the E2F1 transcription factor, which is encoded by a miR-181b target gene.

**Conclusions:**

These findings suggest that miRNA regulate target genes directly through interactions with both conserved and non-conserved target recognition elements, and can lead to both a decrease and increase in transcript abundance. They also multiply their influence through interaction with transcription factor genes exemplified by the observed miR-181b/E2F1 relationship.

## Background

MicroRNA (miRNA) encompass a highly conserved class of endogenous small non-coding RNA which function as the nucleic acid-based specificity factor in post-transcriptional gene silencing (PTGS) [1-3]. In this process miRNA direct a ribonucleoprotein effector complex, known as the RISC, to complementary miRNA recognition elements (MREs) within the 3′ untranslated region (3′-UTR) of mRNA transcripts. In animals these MREs are only partially complementary to the miRNA, with binding to only the miRNA 'seed sequence' - spanning bases 2–7 from its 5′ end - sufficient to mediate PTGS. Due to the redundancy inherent with partial recognition, each miRNA is estimated to target at least 200 genes, to the effect that more than 60% of the human genome could be regulated by miRNA function [4-6]. Not surprisingly, miRNA have been implicated in almost all biological processes including cellular growth, differentiation, apoptosis, and even neurodevelopmental processes such as dendritic spine development and synaptic plasticity [7,8].

While the sequence-specificity of PTGS can be effectively predicted *in silico* using bioinformatic algorithms, the tolerance for partial complementarity in miRNA-MRE interactions can be difficult to reconcile and leads to a relatively high false discovery rate. In an attempt to counter this, many algorithms such as TargetScan [9] and miRBase [10] utilise a filter which can limit predicted MREs to sequences with high levels of conservation. This stringency however, comes at a cost of disregarding MREs that may be specific to particular species or those that have evolved more recently. An additional filter is also often applied to the interpretation of expression data through the assumption that miRNA-mRNA interactions are associated with a reduction in target stability and steady state transcript levels. While this relationship is supported at both the protein [11-13] and RNA [14-16] level, and more recently in experiments investigating both mRNA target levels and their translation [17], there may be alternative modalities for some RISC associated transcripts, beyond gene suppression, that could give rise to discrete functions during development, particularly in highly specialized cells such as neurons. This hypothesis is supported by reports of miRNA stabilising their mRNA targets and even positively regulating gene expression [18-21]. More recently a mechanism for positive transcript regulation though miRNA-mRNA interactions was proposed [22] whereby, one miRNA target is modulated in response to the expression of another by acting as a miRNA sponge. The existence of these competing endogenous RNA (ceRNA) and their influence on miRNA is supported in a number of recent studies [23-26].

In view of these and other possible influences of miRNA on the transcriptome, we established a genome-wide survey of miRNA-associated target-transcript abundance to determine the genomic response to bidirectional modulation of miR-181b and miR-107, both of which have previously been reported to be upregulated in schizophrenia [27,28]. This provided the means to investigate the biology of these miRNA in a number of mammalian cell lines, without assumptions about miRNA binding and mechanism of action. This revealed important regulatory roles for miR-181b in the regulation of the cell cycle, differentiation, and neurodevelopmental processes; and provided support for alternative RISC function, where miRNA-mRNA interactions lead to increases in transcript abundance in addition to the well-documented silencing-associated decrease.

## Results

### Predicted miR-181b target genes and functional annotation

**Figure 1:**
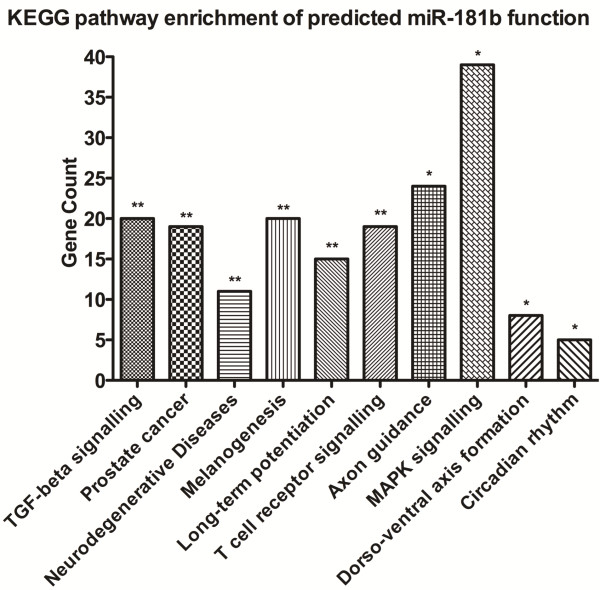
**KEGG pathways analysis of predicted miR-181b target genes.** KEGG pathways analysis of predicted target genes for miR-181b revealed ten significantly enriched pathways: TGF-beta signalling (p=0.0023); prostate cancer (p=0.0037); neurodegenerative diseases (p=0.0061); melanogenesis (p=0.0062); long-term potentiation (p=0.0070); T-cell receptor signalling (p=0.0087); axon guidance (p=0.0106); MAPK signalling (p=0.0217); dorso-ventral axis formation (p=0.0236); and circadian rhythms (p=0.0410). MAPK: mitogen-activated protein kinase. Predicted target genes for miR-181b were generated from the miRGen database and submitted for pathways analysis to DAVID.

The miR-181b predicted target genes were determined using multiple search algorithms in the miRGen database. Functional significance of miR-181b was inferred from pathways analysis of its predicted target genes using the DAVID bioinformatics functional annotation tool (Figure 1). This approach revealed ten significantly enriched pathways (p<0.05), including TGF-beta signalling, neurodegenerative diseases, long-term potentiation, axon guidance, MAPK signalling, and dorso-ventral axis formation (see Additional file 1: Tables S2–S6 for all miR-181b enriched KEGG pathways analyses, p-values, and associated genes).

### Biological processes affected by increased miR-181b expression in cell culture

**Figure 2:**
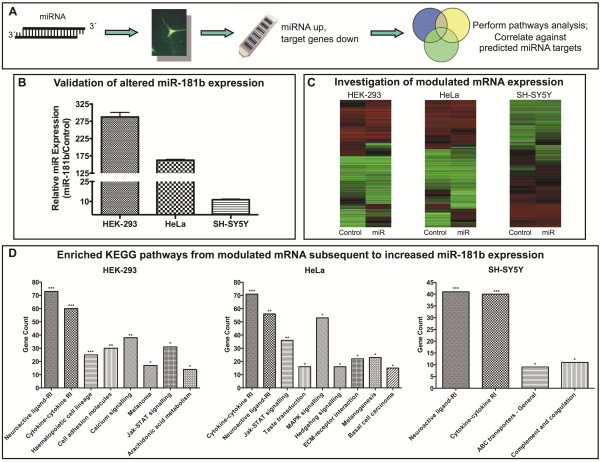
**Biological processes affected by miR-181b over-expression in cell culture via miR-181b transfection.** Panel **A** demonstrates the experimental design for the identification of genes subject to PTGS by increased miRNA concentrations. Canonical miRNA function results in a subsequent decrease in mRNA expression levels detected by whole-genome expression analysis using microarrays. These differentially expressed genes are subsequently utilised for DAVID pathways analysis and correlated against predicted miRNA targets. Panel **B** shows the increase in miR-181b expression levels in comparison to controls for HEK-293, HeLa and SH-SY5Y cell types. Panel **C** shows a clustered-by-gene heat map from whole genome expression microarray data from each cell model, with n=2 per condition. Panel **D** shows the significantly enriched KEGG pathways for each cell type in response to increased intracellular miR-181b levels. RI: receptor interaction; ECM: extracellular matrix; MAPK: mitogen-activated protein kinase.

Cells were transfected with synthetic miR-181b, resulting in a substantial 288-, 165-, and 11.3-fold increase in miR-181b expression in HEK-293, HeLa, and SH-SY5Y cells respectively (Figure 2B). Whole-genome expression analysis was subsequently performed to identify genes altered in the presence of increased intracellular miR-181b concentrations (Figure 2C). In HEK-293 cells this approach identified 3798 differentially expressed genes and eight significantly enriched gene pathways (KEGG), including haematopoietic cell lineage, cell adhesion molecules, and the calcium signalling pathway. Similarly in HeLa cells, 3976 genes and nine significantly enriched pathways were identified, including MAPK signalling and extracellular matrix interaction. In SH-SY5Y cells, 1492 genes and four pathways were significantly enriched, including the ATP binding cassette transporter pathway. Interestingly, neuroactive ligand-receptor interaction and cytokine-cytokine receptor interaction were significantly enriched in all three cell types (Figure 2D).

### Biological processes affected by miR-181b depletion in cell culture

**Figure 3:**
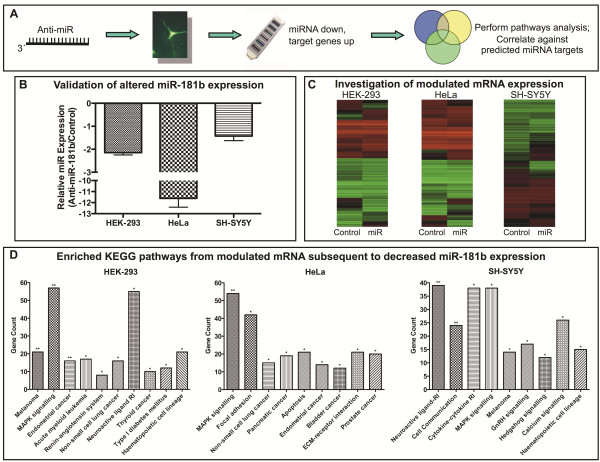
**Biological processes affected by inhibition of endogenous miR-181b in cell culture in response to anti-miR-181b transfection.** Panel **A** illustrates the experimental design for the identification of genes subject to de-repression of PTGS by decreased endogenous miRNA concentrations. Genes elevated in response to a fall in miRNA were utilised for pathways analysis and correlated against predicted miRNA targets. Panel **B** shows the decrease in miR-181b expression levels in comparison to controls for HEK-293, HeLa and SH-SY5Y cell types. Panel **C** shows a clustered-by-gene heat map from whole genome expression microarray data from each cell model, with n=2 per condition. Panel **D** shows the significantly enriched KEGG pathways for each cell type in response to decreased intracellular miR-181b levels.

Cells were transfected with a sequence-specific antisense inhibitor of miR-181b (anti-miR-181b) causing a decrease in the intracellular concentration of miR-181b in the order of 2.2-, 11.6-, and 1.4-fold in HEK-293 cells, HeLa cells, and SH-SY5Y cells respectively (Figure 3B). To characterise the change in mRNA transcript abundance in response to this reduction of endogenous miR-181b, we again used whole genome expression array analysis (Figure 3C). This approach identified 2905 differentially expressed genes and ten significantly enriched gene pathways (KEGG) in HEK-293 cells, while nine pathways were identified in both HeLa and SH-SY5Y cells, along with 2600 and 1782 differentially expressed genes, respectively. The MAPK signalling pathway was significantly enriched in all three cell types, while five pathways were significantly enriched in two of the three cell types, including neuroactive ligand-receptor interaction and haematopoietic cell lineage (Figure 3D).

### Biological processes enriched after bidirectional modulation of miR-181b expression

**Figure 4:**
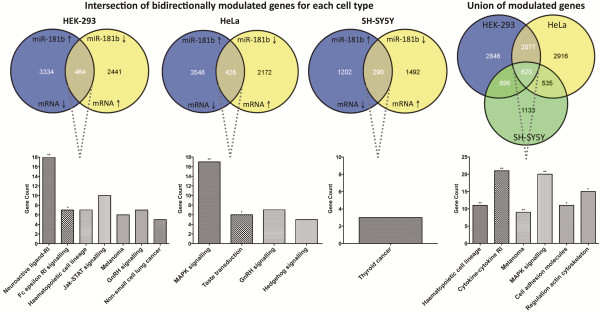
**Analyses of bidirectionally modulated genes in multiple cell types.** The intersection of bidirectionally-modulated genes identifies genes modulated by both increased miR-181b expression (miR treatment) and miR-181b inhibition (anti-miR-181b treatment) in each cell type. Genes modulated by either miR-181b over-expression or inhibition were considered for the union of modulated genes across multiple cell types. The subsequent KEGG pathways analyses on these genes of interest revealed significantly enriched pathways, as evident in the bottom half of this figure.

For a more stringent appraisal of genes and processes influenced by miR-181b expression, we examined genes both downregulated in response to miR-181b over-expression and upregulated by inhibition of endogenous miR-181b using the anti-miR inhibitor. This revealed 464, 428, and 290 genes bi-directionally modulated in HEK-293, HeLa, and SH-SY5Y cells respectively (Figure 4). KEGG pathways analysis on these genes revealed a statistically significant enrichment of genes involved in neuroactive ligand-receptor interaction and Fc epsilon receptor I signalling in HEK-293 cells; and MAPK signalling and taste transduction in HeLa cells. No significantly enriched pathways were identified in SH-SY5Y cells. To identify target genes common to each cell type, our analysis was expanded to genes modulated by either miR-181b over-expression or inhibition. In doing so, we observed 620 genes altered across all three cell types, with six significantly enriched pathways: haematopoiesis, cytokine-cytokine receptor interaction, melanoma development, MAPK signalling, cell adhesion molecules, and regulation of actin cytoskeleton.

### Correlation between miRNA–associated gene expression and target prediction

#### Comparison of miRNA over-expression and inhibition

**Figure 5:**
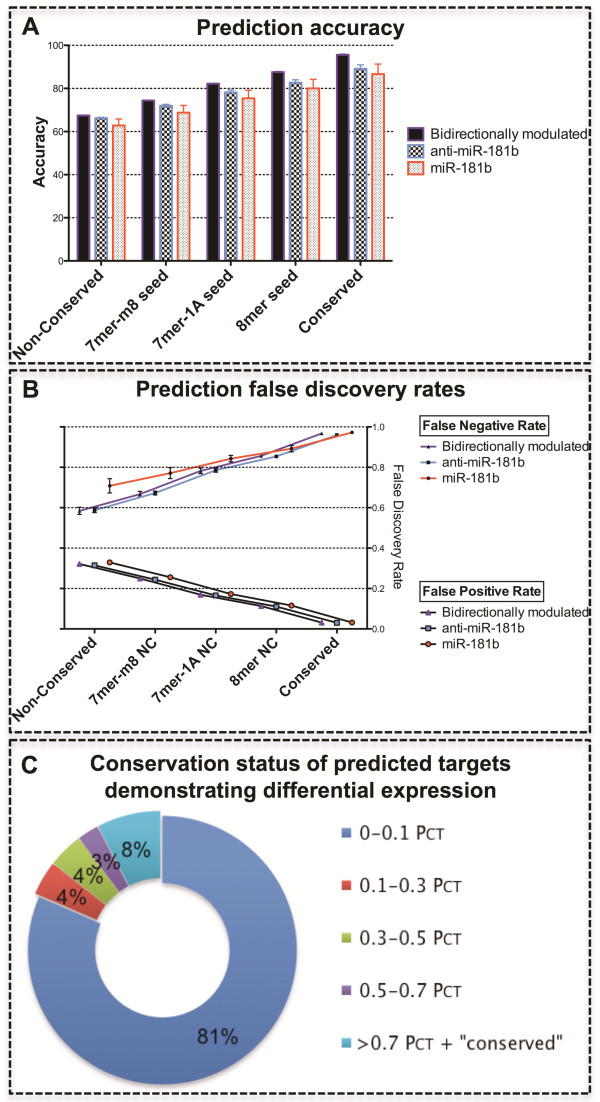
**The performance of conserved and non-conserved target predictions across multiple biological datasets.** Panel **A** illustrates the accuracy with which modulated genes were correctly predicted as either targets or non-targets by Targetscan. Error bars were calculated using the mean, N, and standard deviation across HEK-293, HeLa, and SH-SY5Y datasets. Panel **B** illustrates the false discovery rates associated with Targetscan’s prediction of genes modulated subsequent to altered miRNA expression. A false negative indicates a gene differentially expressed with miRNA modulation, but not a predicted miR-181b target; and a false positive indicates a predicted miR-181b target that is not differentially expressed with miRNA modulation. Error bars were calculated using the mean, N, and standard deviation across HEK-293, HeLa, and SH-SY5Y datasets. Panel **C** shows the conservation status of predicted target genes modulated in response to altered miR-181b expression. The values in this figure represent the average values across both miR-181b over-expression and inhibition in HEK-293, HeLa, and SH-SY5Y cell types. PCT: Probability of conserved targeting; the lower the probabilistic value, the poorer the conservation of the predicted binding site across multiple species.

To further investigate observed changes in response to miRNA modulation, the Targetscan algorithm was used as a framework to measure various prediction parameters. In comparing our biological results with Targetscan’s predictions, a criterion of accuracy was calculated to determine the proportion of genes correctly predicted to respond as either targets or non-targets (Figure 5A). Repeated measures ANOVA (rmANOVA) revealed a significant difference in accuracy between models of miRNA over-expression; inhibition; and bidirectional modulation (p<0.0001). Bidirectional modulation provided the greatest average accuracy across each cell type for Targetscan’s various prediction parameters of conservation and seed region (81.5%); significantly higher than miR-181b inhibition (77.6%, p<0.0001); which was in turn significantly higher than miR-181b over-expression (74.7%, p=0.0006).

The false-positive discovery rate (FPR) was also calculated to indicate the proportion of predicted targets that were not differentially expressed in response to miRNA modulation (Figure 5B). This was significantly different (rmANOVA) for miRNA over-expression, inhibition, and bidirectional modulation across each cell type and prediction parameter (p=0.0046). In a similar fashion, the false-negative discovery rate (FNR) was calculated to determine the proportion of genes that were differentially expressed upon modulation of miRNA expression, despite not being predicted by Targetscan to be regulated by miR-181b (Figure 5B). While this may include genes differentially expressed by non-miRNA influences as a result of the transfection process, it can also give an indication of genes that may be influenced secondary to miRNA function, downstream in a signalling pathway from a gene that is a direct miRNA target. There was also a significant difference between the mean FNR for miR-181b over-expression, inhibition, and bidirectional approaches (p=0.0067), with average FNRs for miRNA inhibition and bidirectional modulation (0.77) significantly lower than for miRNA over-expression (p<0.009).

#### Influence of cell lineage

The prediction-response accuracy to miRNA modulation was significantly different in different cell types (rmANOVA, p<0.0001) (Figure 5A). The SH-SY5Y cell type provided the greatest accuracy (79.8%); significantly higher across Targetscan’s various prediction parameters of conservation and seed region than HeLa (77.1%, p=0.0049) and HEK-293 (77.0%, p<0.0001) cells. There was no significant difference in accuracy between HEK-293 and HeLa cells; these data sets were highly similar with a correlation coefficient of 0.997 (p<0.0001). There was also no significant difference in the FNR (p=0.6143) or FPR (p=0.1630) between cell types (Figure 5B).

#### Influence of seed region

To explore the influence of seed region composition in the prediction of observed changes upon miRNA modulation, Targetscan’s non-conserved predictions were categorised by their length and composition of seed region (Figure 5A and B). The 8mer seed sequence classification demonstrated the greatest prediction-response accuracy (83.4%); significantly higher on average across all experimental parameters than 7mer-1A (78.6%, p<0.0001); which itself predicted significantly better than the 7mer-m8 region (71.7%, p<0.0001). For FPRs, the 8mer seed region offered the lowest FPR (0.11); significantly lower than 7mer-1A (0.17, p<0.0001); which was in turn significantly lower than 7mer-m8 (0.25, p<0.0001). Accordingly, the FNR of the 8mer seed (0.87) was significantly higher than 7mer-1A (0.80, p<0.0001), which was in turn significantly higher than 7mer-m8 (0.70, p<0.0001).

#### Influence of target sequence conservation

In each cell condition, the predicted miR-181b target-response accuracy was significantly improved from 65.5% to 90.5% (p<0.0001) when excluding non-conserved targets from these analyses and considering only conserved targets (Figure 5A). While this was reflected in a substantial decrease in FPR from 0.32 to 0.03 (p<0.0001), it was also accompanied by an even greater increase in FNR from 0.63 to 0.97 (p<0.0001) (Figure 5B). The influence of conservation on the FNR was highlighted by the observation that on average 81% of modulated genes had a probability of conserved targeting score (P_CT_) of less than 0.1 (Figure 5C; Additional file 2: Figure S1).

### Validation of MREs identified in differentially expressed target genes

**Figure 6:**
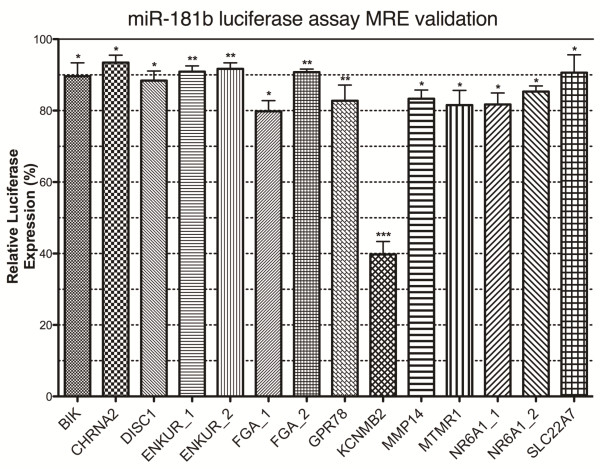
**Reporter gene analysis of miRNA recognition elements (MRE).** Putative MREs from genes modulated by miRNA expression were cloned into the 3^′^-UTR of the firefly luciferase gene in pMIR-REPORT. Responsiveness of the firefly luciferase reporter gene to increased miR-181b expression (miR-181b transfection) was analysed with respect to a pRL-TK renilla luciferase control. Data was normalised against mutant miR-181b miRNA control transfection. This data was obtained from n=4 experiments, each performed in triplicate, and analysed using a one-tailed T-test.

Genes sensitive to both increased and decreased miRNA expression in multiple cell lines were scanned for potential miR-181b MREs using the miRanda shell script. A selection of these sites were then cloned downstream of a luciferase reporter gene construct and their interactions with miR-181b examined in transfected cells using a dual luciferase reporter gene assay. This identified 14 novel miR-181b MREs within 11 genes (p<0.05): BCL2-interacting killer (apoptosis-inducing) (BIK); cholinergic receptor, nicotinic, alpha 2 (neuronal) (CHRNA2); disrupted in schizophrenia 1 (DISC1); enkurin (ENKUR/c10orf63); fibrinogen alpha chain (FGA), G protein-coupled receptor 78 (GPR78); potassium large conductance calcium-activated channel, subfamily M, beta member 2 (KCNMB2); matrix metallopeptidase 14 (membrane-inserted) (MMP14); myotubularin related protein 1 (MTMR1); nuclear receptor subfamily 6, group A, member 1 (NR6A1); and solute carrier family 22 (organic anion transporter), member 7 (SLC22A7) (Figure 6; Table 1).

**Table 1:** miR-181b luciferase assay MRE validation

**Gene**	**Fold change**	**p-value**	**MRE**
BIK	−10.38%	0.0187	ATTCCGAGGAGCAGGAGTGCTC
CHRNA2	−6.54%	0.0482	CACTGGCTGGAGAGCAACGTGGATGCC
DISC1	−8.55%	0.0370	TCTAGTTCATTAAAAGTGAATGTT
ENKUR_1	−9.10%	0.0014	CCTTAATGAATAAAGTAATGGATCGTA
ENKUR_2	−8.33%	0.0010	CATCGCTAAGTAAGCAACTTAAGTTGCTT
FGA_1	−20.19%	0.0225	TCCACTAGACGTTGTAATGCACACT
FGA_2	−9.24%	0.0025	TTTGATCCAGCAAAGAATGGATGGATC
GPR78	−17.25%	0.0032	GACGCCCAAAGCAGGATGTGTCTT
KCNMB2	−60.23%	<0.0001	CATTACCTGTGAGCTGACTGAATGTT
MTMR1	−18.45%	0.0496	CCCCTGGCTGACTAGGACTGTT
MMP14	−16.68%	0.0490	CCCACCCAGCCCACCCATTGAAGTCT
NR6A1_1	−18.27%	0.0152	TTCACGACAGAGTTGAATGTAT
NR6A1_2	−14.68%	0.0180	ACCAGCTGAGCAGAATGCCATGTT
SLC22A7	−9.38%	0.0392	CACCCTGCAGGGCAATGCATGTC

### Investigation of modulated genes devoid of miR-181b MREs

To investigate the potential influence of transcription factors in differentially expressed genes devoid of miR-181b binding sites, we analysed their transcription factor motif composition using the TRANSFAC database. This revealed a consistent and highly significant enrichment of genes containing recognition signatures for several core transcription factors across each condition and cell type, including E2F transcription factor 1 (E2F1), the ETS domain transcription factors E74-like factor 1 (ELF1) and ETS-like gene 1 (ELK1), and the early growth response (KROX) transcription factor family; all of which possess miR-181b predicted binding sites. The E2F transcription factor 1 (E2F1) was particularly significant with multiple predicted miR-181b MREs and repeated enrichment of E2F1 transcription factor recognition signatures across multiple conditions (Additional file 3: Figure S2).

**Figure 7:**
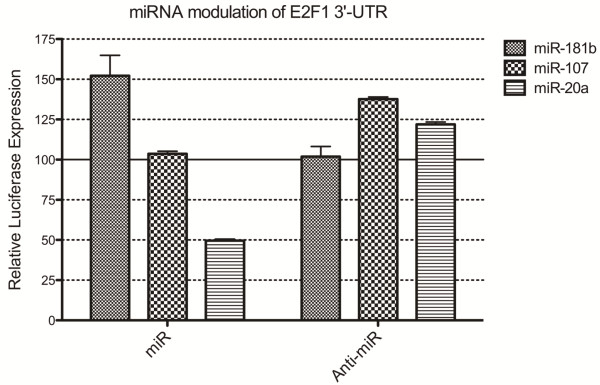
**miRNA-mediated regulation of E2F1 3**^′^**-UTR reporter gene expression.** The sensitivity of the E2F1 3^′^-UTR to intracellular 181b, miR-107, and miR-20a levels was determined by luciferase reporter gene expression in the presence of either synthetic miRNA or corresponding anti-miR inhibitor. In each case the response was normalised against the respective miRNA and anti-miR control oligos. This data was obtained from n=4 experiments, each performed in triplicate.

To investigate the possibility that miR-181b is regulating E2F1 in these cells, a reporter gene containing the E2F1 3′-UTR was co-transfected with miR-181b or its anti-miR inhibitor (Figure 7). As expected we observed a significant (p<0.0001) miR-181b associated change in luciferase activity, however, the direction was contrary to expectation with a 52% increase in E2F1 reporter gene expression in response to miR-181b over-expression. To confirm that this response was not a reporter gene artefact, other E2F1 targeting miRNA miR-107 and miR-20a were also transfected and analysed. These both produced more conventional inversely proportional relationships with the miR-107 inhibition elevating reporter expression 37% (p<0.0001). Similarly, miR-20a over-expression caused 50% suppression (p<0.0001) while miR-20a inhibition produced a 22% increase (p=0.0009). This demonstrates that miR-181b has the capacity to modulate E2F1 expression through it’s 3′-UTR, and suggests a mechanism to explain miR-181b associated changes in genes lacking a corresponding MRE.

### Bidirectionally modulated genes are enriched with miR-181b and E2F1 binding sites

**Figure 8:**
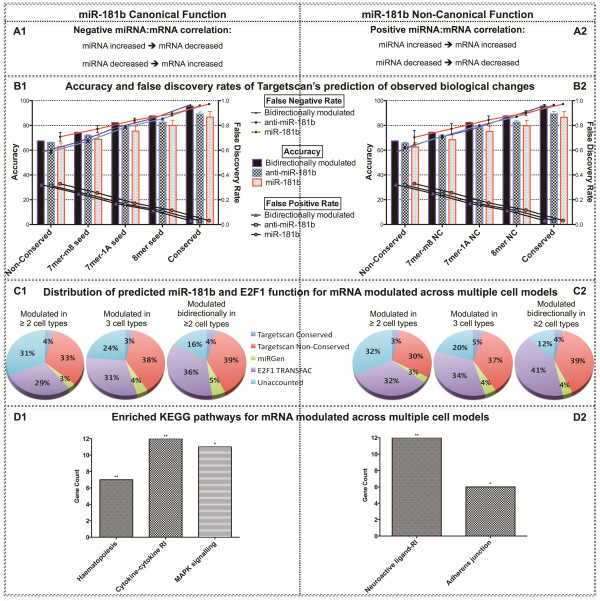
**Comparison of canonical (left) and non-canonical (right) miRNA-mRNA relationship.** Panel **A**, scheme. Panel **B** contains charts of accuracy and false discovery rates associated with Targetscan’s prediction of observed changes in mRNA expression. Panel **C**, pie charts illustrating the distribution of miR-181b and E2F1 target genes predicted using different algorithms and parameters in multiple cell types. Signal-to-noise ratio is shown to increase for both canonical and non-canonical function as stringency increases from genes modulated by either miR-181b over-expression or inhibition across at least two cell types; to genes modulated by either miR-181b over-expression or inhibition across all three cell types; to genes modulated by both miR-181b over-expression and inhibition across at least two cell types. Panel **D** contains charts of enriched KEGG pathways from genes modulated by either miR-181b over-expression or inhibition across at least two cell types. RI: receptor interaction. MAPK: mitogen-activated protein kinase.

While a large proportion of miR-181b associated changes can be attributed to the presence of corresponding MREs or E2F1 binding motifs (52% in HEK-293 and HeLa cells; 70% in SH-SY5Y cells), this proportion increases significantly to over 80% in HEK-293 and HeLa cells when only bidirectionally modulated genes are considered (Additional file 4: Figure S3). Further stringency of this prediction could also be attained by restricting the analysis to genes changed by both miR-181b over-expression and inhibition in two or more cell types, with miR-181b MREs alone accounting for 48% of differentially expressed genes, and MRE plus E2F1 motifs covering 84% (Figure 8C1).

### Positively correlated miRNA-mRNA interactions

While the transcripts of miRNA target genes are generally expected to be reduced by their corresponding miRNA and display an inverse relationship, it is possible that some interactions, exemplified by our E2F1 reporter gene, may not display this behaviour. To explore this possibility further we investigated genes displaying a positive miRNA-mRNA correlation rather than the canonical negative miRNA-mRNA correlation. Interestingly, we observed very similar statistics for both types of interactions with regards to the relationship between gene expression and target prediction for the direction of miR-181b modulation; cell lineage; target conservation; and seed sequence (Figure 8B; Table 2). The only parameter not representing a significant parallel between canonical and non-canonical response was the FNR for conserved targets, though a paired student’s t-test reveals no significant difference (p=0.76). Moreover, predicted miR-181b and E2F1 function for both canonical and non-canonical responses was also highly correlated (R^2^: 0.990, p<0.0001) in classifying the contribution to the gene expression profile across all conditions. Again, more stringent analysis of genes modulated in multiple conditions and cell types was characterised by an increase in the proportion of observed changes that can be attributed to primary and downstream miR-181b activity (Figure 8C2; Additional file 4: Figure S3).

**Table 2:** Summary of miR-181b correlation analyses for canonical and non-canonical miRNA-mRNA outcomes

	**Accuracy**	**FPR**	**FNR**
**miR Modulation**			
miR	R^2^: 0.995; p<0.0001	R^2^: 1.000; p<0.0001	R^2^: 0.976; p<0.0001
anti-miR	R^2^: 0.994; p<0.0001	R^2^: 1.000; p<0.0001	R^2^: 0.985; p<0.0001
Bidirectional	R^2^: 1.000; p<0.0001	R^2^: 1.000; p<0.0001	R^2^: 0.970; p<0.0001
**Cell Type**			
HEK-293	R^2^: 0.996, p<0.0001	R^2^: 1.000, p<0.0001	R^2^: 0.988, p<0.0001
HeLa	R^2^: 0.996, p<0.0001	R^2^: 1.000, p<0.0001	R^2^: 0.983, p<0.0001
SH-SY5Y	R^2^: 1.000, p<0.0001	R^2^: 1.000, p<0.0001	R^2^: 0.989, p<0.0001
**Conservation**			
Conserved	R^2^: 0.986, p<0.0001	R^2^: 0.751, p=0.0196	R^2^: 0.193, p=0.6183
Non-Conserved	R^2^: 0.959, p<0.0001	R^2^: 0887, p=0.0014	R^2^: 0.835, p=0.0051
**Seed Sequence**			
8mer	R^2^: 0.981, p<0.0001	R^2^: 0.803, p=0.0091	R^2^: 0.803, p=0.0092
7mer-m8	R^2^: 0.970, p<0.0001	R^2^: 0.853, p=0.0034	R^2^: 0.790, p=0.0112
7mer-1A	R^2^: 0.975, p<0.0001	R^2^: 0.780, p=0.0131	R^2^: 0.782, p=0.0127

### Genome-wide analysis of miR-107 associated gene expression

**Figure 9:**
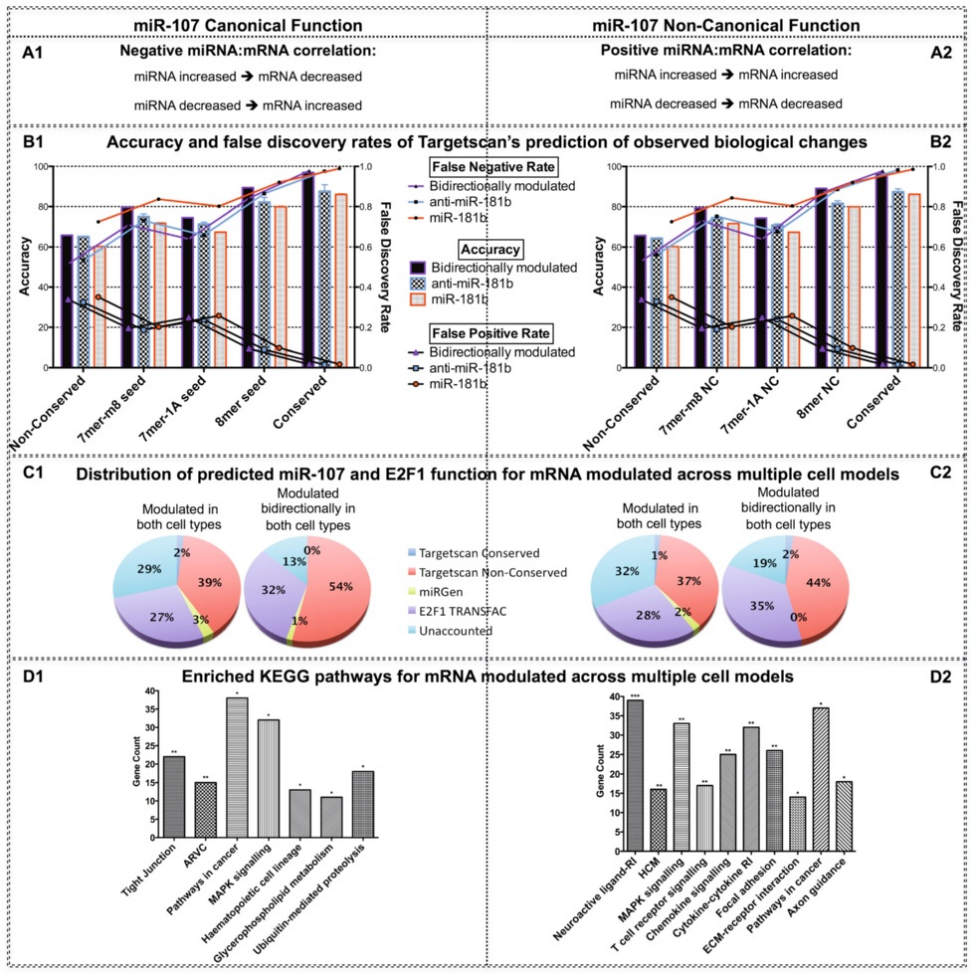
**Comparison of canonical (left) and non-canonical (right) miR-107 function.** Figure legend as per Figure 8 except in respect to miR-107.

To further investigate the influence of miRNA on the transcriptome, we also investigated the bidirectional modulation of miR-107 in HEK-293 and HeLa cells (Additional file 5: Figure S4; Additional file 1: Tables S7–S10). Overall, the gene expression analysis of canonical miR-107 function demonstrated great consistency with miR-181b in respect to prediction-response evaluation using a Targetscan framework (Figure 9B1). There was also a significant difference (p=0.0002) in the accuracy of the algorithm to predict the observed changes in gene expression for miR-107 over-expression, inhibition, and bidirectional conditions. In both HEK-293 and HeLa cell types, bidirectionally modulated genes provided the greatest accuracy, significantly higher than miRNA inhibition (p=0.0015); which was in turn significantly higher than miRNA over-expression (p=0.0006). The FPR was significantly lower in miRNA inhibition (p=0.0027) than bidrectionally modulated; which was in turn significantly lower than for miRNA over-expression (p=0.0013). The FNR was lowest with bidirectional modulation, followed by miRNA inhibition (p=0.0042), with miR-107 over-expression providing the highest FNR (p=0.0009).

Whilst there was no significant difference in the accuracy between HEK-293 and HeLa cell types (p=0.1268), the FPR (p=0.0202) and FNR (p=0.0095) for HEK-293 cells were significantly lower than in HeLa cells. In considering conservation parameters, Targetscan’s conserved parameter showed a significantly greater accuracy than non-conserved (p<0.0001); also providing a significantly lower FPR (p<0.0001) and higher FNR (p=0.0002). Furthermore, of the predicted targets sub-classified according to seed region, 8mer offered the greatest accuracy, significantly higher than 7mer-m8 (p<0.0001), which was in turn higher than 7mer-1A (p<0.0001); FPR was significantly lowest for 8mer (p<0.0001), followed by 7mer-1A, with 7mer-m8 yielding the highest FPR (p<0.0001); conversely, the FNR was significantly higher in 8mer than 7mer-m8 (p=0.0002), which was in turn significantly higher than the 7mer-1A (p=0.0013) seed region. With miR-107 also possessing the capacity to regulate E2F1 expression, the proportion of observed changes that can be attributed to primary and secondary miR-107 function were also investigated, explaining 87% of bidirectionally modulated genes in both cell types (Figure 9C1; Additional file 4: Figure S3).

Non-canonical miR-107 interactions were also investigated and compared with conventional interactions (Figure 9; Table 3). As with miR-181b, the only parameter not to show significant correlation between canonical and non-canonical miR-107 function is for Targetscan’s conserved parameter, in which the FPR and FNR were not significantly correlated, despite no significant difference between these features by t-test (FPR: p=0.7441; FNR: p=0.7222). All remaining parameters for analysis demonstrated highly significant correlation between canonical and non-canonical miR-107 function (Table 3). Furthermore, investigating miR-107 and E2F1 predicted function to explain genomic changes in the non-canonical direction revealed a highly significant correlation between canonical and non-canonical responses (R^2^: 0.994, p<0.0001) across all miRNA modulation conditions. Again, combining multiple conditions provided a more accurate prediction of observed responses, with 81% of genes bidirectionally modulated in both cell types (Figure 9C2; Additional file 4: Figure S3).

**Table 3:** Summary of miR-107 correlation analyses for canonical and non-canonical miRNA-mRNA outcomes

	**Accuracy**	**FPR**	**FNR**
**miR Modulation**			
miR	R^2^: 1.000, p<0.0001	R^2^: 1.000, p<0.0001	R^2^: 0.997, p<0.0001
anti-miR	R^2^: 0.996, p<0.0001	R^2^: 1.000, p<0.0001	R^2^: 0.999, p<0.0001
Bidirectional	R^2^: 1.000, p<0.0001	R^2^: 1.000, p<0.0001	R^2^: 0.992, p<0.0001
**Cell Type**			
HEK-293	R^2^: 0.999, p<0.0001	R^2^: 1.000, p<0.0001	R^2^: 0.998, p<0.0001
HeLa	R^2^: 0.999, p<0.0001	R^2^: 1.000, p<0.0001	R^2^: 0.997, p<0.0001
**Conservation**			
Conserved	R^2^: 0.990, p=0.0002	R^2^: 0.675, p=0.1417	R^2^: 0.519, p=0.2919
Non-Conserved	R^2^: 0.990, p=0.0001	R^2^: 0.976, p=0.0008	R^2^: 0.980, p=0.0006
**Seed Sequence**			
8mer	R^2^: 0.993, p<0.0001	R^2^: 0.875, p=0.0224	R^2^: 0.955, p=0.0030
7mer-m8	R^2^: 0.994, p<0.0001	R^2^: 0.968, p=0.0016	R^2^: 0.980, p=0.0006
7mer-1A	R^2^: 0.996, p<0.0001	R^2^: 0.971, p=0.0012	R^2^: 0.980, p=0.0006

### Functional annotation of non-canonical miRNA-mRNA interactions

A comparative pathways analysis was performed to investigate the functional characteristics between genes exhibiting canonical (negative miRNA-mRNA correlation) and non-canonical (positive miRNA-mRNA positive correlation) responses across all three cell types subsequent to miR-181b modulation (Figure 8D). Genes with a canonical response were significantly enriched in haematopoietic cell lineage, cytokine-cytokine receptor interaction, and MAPK signalling; whereas those displaying a non-canonical response were significantly enriched in the neuroactive ligand-receptor interaction and adherens junctions pathways. This comparative analysis was also applied to the miR-107 dataset to identify genes modulated across both the HEK-293 and HeLa cell types (Figure 9D), with canonical function showing an enrichment in pathways including tight junction, arrhythmogenic right ventricular cardiomyopathy, pathways in cancer, MAPK signalling, and haematopoietic cell lineage; whilst non-canonical function revealed enrichment in pathways including neuroactive ligand receptor interaction, hypertrophic cardiomyopathy, MAPK signalling, T cell receptor signalling, pathways in cancer, axon guidance, and the mTOR signalling pathway.

## Discussion

In this investigation we considered four key epistemic concepts essential to understanding miRNA function. Firstly, we theorised that the function of a miRNA is more than the sum of its targets, and factored into our investigation the potential for miRNA target gene regulation to produce secondary effects downstream from the direct target, and that such effects constitute an important contribution to the biological function of the miRNA. Secondly, we considered the notion that the biology of a miRNA is influenced by its expression, and sought to bidirectionally modulate miRNA levels to gain insight into miRNA function at both endogenous levels and under conditions of increased expression. The third consideration was to investigate miRNA function in multiple cell types to gain insight into how specific cellular environments may influence the profile of genes a specific miRNA may or may not have the capacity to influence. Finally, rather than considering miRNA as direct mediators of gene silencing, we adopted an ontological perspective of miRNA as sequence-specificity guides for protein complexes to in turn regulate gene expression. As such, we objectively investigated both negative and positive miRNA-mRNA expression patterns and evaluated this against an established target prediction framework to gain a more complete understanding of miRNA function.

### miR-181b functions as a complex regulator of cell cycle progression and differentiation

There is an ever-increasing body of evidence linking miRNA-mediated gene dysregulation to various pathophysiological processes. However, apart from a select number of validated miRNA target genes – considering each miRNA is predicted to regulate potentially hundreds of target genes – the biological function of miRNA in different cellular environments is poorly understood due to their vast functional pleiotropy. This is exemplified by miR-181b which has been shown to be associated with schizophrenia [28]; muscle development [29]; haematopoiesis [30-33]; and a variety of cancers, both as an oncogene [34-43] and a tumour suppressor [44-49]. In this study we employed an empirical genomics approach to miRNA target analysis through the generation of bidirectional transfection models in HeLa, HEK-293 and SH-SY5Y cell types. Genes differentially expressed in response to elevation or repression of miRNA expression were used to define the biology of the miRNA and ascertain both primary target genes and their downstream effects.

We observed a large number of genes in the haematopoietic cell lineage pathway that were differentially expressed with increased intracellular miR-181b concentrations in HEK-293 cells, suggesting that genes within the haematopoietic pathway can be regulated by miR-181b when this miRNA is over-expressed in this cell line; as well as by endogenous miR-181b concentrations in both HEK-293 and SH-SY5Y cells. This supports the strong and repeated association of miR-181b and haematopoiesis previously reported in the literature, with targets including the murine Bcl-2, CD69 and the T-cell receptor [32], along with the chemokine [C-X-C motif] receptor 4 (CXCR4) [33].

In each cell type we also observed a repeated enrichment of modulated genes involved in various pathways associated with oncogenesis. Firstly, the inhibition of miR-181b function revealed an enrichment of modulated genes in the MAPK signalling pathway for HEK-293, HeLa and SH-SY5Y cells, suggesting that endogenous miR-181b – directly or indirectly – regulates this critical cell-cycle signalling pathway in each of these cell types; pathways analysis revealed that only in HeLa cells did miR-181b appear to have the capacity to extend its regulation on this signalling pathway at higher miRNA concentrations. Further to this, we observed an enrichment of modulated genes involved in cytokine-cytokine receptor interaction by endogenous miR-181b levels in SH-SY5Y cells, and by increased miRNA concentrations in HEK-293 and HeLa cells. In the latter of the two cell types, increased miR-181b levels also saw the modulation of the JAK-STAT signalling pathway, while endogenous miRNA expression was associated with regulating pathways involved in endometrial cancer, focal adhesion, and extracellular-matrix interaction. A complex association of both positive and negative regulation of oncogenic processes was also suggested by the identification of a miR-181b MRE within the pro-apoptotic protein BIK [50], as well as a highly-conserved MRE in the tumour invasion factor MMP14 [51,52]. Both of these interactions were supported by reporter gene assay. Similarly a conserved miR-181b MRE was also identified in MTMR1, previously identified as an important regulator of myogenesis through its association with muscular disorders such as myotubular myopathy and congenital myotonic dystrophy [53,54], though its exact biological role is still unclear [54]. Positive regulation of myogenesis has also been supported by miR-181b suppression of HOXA11 and a HOXA11 reporter gene [29].

Importantly, we observed a consistent and significant enrichment of the neuroactive ligand-receptor interaction pathway across all three cell types treated with miR-181b, in addition to its enrichment in both HEK-293 and SH-SY5Y cells treated with anti-miR-181b. This was further supported by the validation of miR-181b MREs within the 3′-UTRs of the schizophrenia susceptibility genes DISC1 [55-57], ENKUR [58] and GPR78 [59], as well as the nicotinic acetylcholine receptor CHRNA2, and the potent binding site in KCNMB2, involved in controlling neuronal excitability by functioning as a subunit in large conductance voltage and calcium−activated potassium channels – also known as BK, MaxiK, or Slo channels [60,61]. These interactions have significant implications for schizophrenia as miR-181b has been shown to be altered in the disorder [27,28].

These results, in addition to the literature, indicate a key role for miR-181b in the fine-tuning of expression levels of numerous functionally related genes in specific signalling pathways. The collective biological influence, while different in the various cell types, appeared to converge in regulation of the cell cycle, differentiation states, and neurodevelopmental processes.

### Reconciling miRNA-associated gene expression with predicted function

While the biological activity of miRNA are complex, the ability to predict their interactions with target mRNA provides important insight into the diverse functions of these molecules. However, these predictions are prone to both substantial under and over-prediction depending on the stringency, and usually fail to account for the local influence of other miRNA, RNA binding proteins and RNA secondary structure, and are incapable of determining downstream effects. To investigate the power and limitations of target prediction we compared miR-181b associated genes *in vitro* with conserved and non-conserved targets predicted by Targetscan. Interestingly, only a relatively small proportion (av. 3.46%) of responsive genes contained conserved 3′-UTR motifs, compared to the significantly larger proportion (av. 34.94%) which had non-conserved miRNA recognition elements. While the conserved target predictions had a lower false positive rate, they displayed a very high rate of false negatives; whereas the inclusion of non-conserved targets provided a substantial improvement in the false negative rate, with some cost in terms of an increase in the false positive discovery rate. This suggests that despite their recent emergence, in an evolutionary context, many non-conserved miRNA elements are likely to exert a significant influence on their host gene.

The seed sequence composition was also reflected in the prediction rate, with the 8mer seed region providing the greatest predictive power for observed changes for each miRNA. The variation observed for the 7mer-1A and 7mer-m8 seed regions between miR-181b and miR-107 supports the notion that determinants for target recognition exist outside of the seed region [62], while also highlighting the increased false-positive rate associated with non-conserved target predictions. The biological relevance of these predictions is indicated by the increased proportion of biological changes explained through primary and secondary miRNA function when considering only those genes modulated across multiple conditions and cell types, thereby supporting the adoption of a broad approach in biological modelling of miRNA function. With respect to *in silico* modelling of miRNA function, it may also be more prudent to accept some degree of over-prediction associated with the acquisition of non-conserved targets genes rather than a more conservative approach using only conserved targets, which appear to be associated with an unacceptably high level of under-prediction.

Collectively modulated genes with predicted miR-181b MREs, both conserved and non-conserved, still only accounted for a proportion (av. 38.59%) of all miR-responsive genes. Significantly, many of these genes lacking predicted MREs for miR-181b contained binding motifs for the E2F1 transcription factor (av. 30.74%). This suggests that miR-181b, predicted to bind to multiple MREs within the 3′-UTR of E2F1, is able to indirectly influence E2F1-regulated genes as a secondary consequence of E2F1’s own regulation by miR-181b. Surprisingly, E2F1 3′-UTR luciferase reporter gene expression was shown to be elevated in the presence of its cognate miRNA, rather than being repressed in accordance with the canonical PTGS mechanism. Despite the unexpected direction of this response, it nevertheless provides a means for this downstream influence observed in response to miR-181b modulation. While there is likely to be other miR-181b target genes with the potential to exert a downstream influence, these should be limited by the relatively short time frame of this experiment (24 hours). In addition to this influence, there is also a range of other mechanisms that may underlie the discrepancy between observed and predicted miR-181b response. For example, alternative polyadenylation and splicing often produces tissue-specific 3′-UTR variants [63,64], such that specific gene isoforms may contain or exclude MREs for miR-181b. Moreover up to 40% of currently-predicted miRNA target elements reside in regions which may not always be part of the mature mRNA transcript [65]. MREs outside the 3′-UTR, such as the coding regions or even 5′-UTRs [66], are also thought to have some capacity for miRNA mediated gene silencing [67]. Either way, while these genes may not constitute direct targets of miR-181b, they are still significant to understanding its function in the context of specific biological environments.

### miRNA suppression compared with miRNA overexpression

In this study we found that inhibition of endogenous miRNA using anti-miR transfection yielded gene expression changes that reflected all target prediction variables more favourably than miRNA over-expression. This suggests that the subtle modification of physiological levels of endogenous miRNA leads to more significant and biologically indicative changes in target gene expression than the supra-physiological expression induced by synthetic miRNA. One possibility is that target genes are already approaching saturation with endogenously synthesised molecules and there is only a small pool of free targets that become associated with transfected miRNA. Another possibility is that exogenously delivered miRNA compete with endogenous miRNA for RISC association and cause some distortion of the overall gene silencing profile by repressing the function of endogenous miRNA. Interestingly, we also observed elevation of differentially expressed genes with E2F1 motifs after miR-181b over-expression.

### Support for non-canonical miRNA function

miRNA-mRNA interactions are generally thought to result in gene silencing by reducing the stability and translation of RISC-associated mRNA. However, there are some reports to suggest that this is not always the case with miRNA also being capable of protecting or even elevating the steady state levels of their RISC-associated transcripts. In support of this hypothesis we identified a substantial group of predicted miRNA target genes which displayed positive correlation with the intracellular miRNA rather than the expected or canonical, inversely proportional response. These target genes displayed the same properties as the negatively correlated targets including their dependency on the direction of miRNA modulation, cellular background, and conservation; in addition to the differential response to predicted seed threshold paring; as well as contributions to secondary effects through predicted E2F1 function; and increased accuracy of target prediction when analysing only genes modulated in multiple treatment/cell type combinations. These observations were also replicated for miR-107 expression profiling, all displaying highly significant correlation between observed canonical and non-canonical responses.

This highly consistent correlation of miRNA target prediction for both canonical and non-canonical miRNA function suggests that there are functionally significant alternative fates for miRNA-associated mRNA. One possibility is that some RISC-associated miRNA/mRNA may be involved more in post-transcriptional trafficking and/or translational silencing, and as a consequence the steady state levels of both molecules are correlated as the mRNA is protected or sequestered through its association with miRNA and other ribonuclear proteins within intracellular compartments. This form of translational control may be important for complex highly-differentiated cells. For example, neurons may have subcellular ribonuclear protein structures that can support this form of functional partitioning that may give rise to these positively correlated interactions [68], and it is therefore interesting that pathways associated with non-canonical target gene response tended to be more enriched for neuronal pathways than canonical function. While the mechanism and features that mark this kind of relationship are yet to be determined, by characterising the genes, miRNA and cell types involved, we may get a clearer picture of the underlying molecular biology. In one hypothesis, miRNA themselves may be the targets of other miRNA and hence the increase of one miRNA may cause a net increase in gene expression because of its repression of another miRNA targeting the same genes. In this respect, miR-181b has been shown to negatively regulate let-7 expression [69]. Our analysis of this effect did not reveal enrichment of predicted let-7 MREs between genes demonstrating negative and positive miRNA-mRNA correlation for miR-181b. Further evidence of this phenomenon in miR-107 datasets suggests this behaviour extends beyond let-7-miR-181b feedback.

Previous investigation of positively-acting miRNA suggest that this feature may be attributed to a cell cycle-dependent switch between translation activation and repression: at certain stages of the cell cycle let-7 was shown to switch from repressor to activator [18], with increased mRNA levels attributed to miRNA binding at AU-rich elements (ARE): elements that promote mRNA degradation. However, an investigation into the ARE distribution identified no pattern specific to positively versus negatively correlated genes, and the ARE in the 3-UTR of E2F1 neither overlaps the binding sites for miR-181b, -107 or -20a.

## Conclusions

In summary, gene expression profiling was used to identify the molecular influence of miRNA and evaluate the fate of associated target transcripts. This revealed that a large proportion of target genes are not conserved and that many genes are modified by miRNA-associated secondary influence, exemplified by the relationship between miR-181b E2F1 transcription factor and genes with E2F1 motifs. The analysis of positively associated target genes also suggested that a substantial proportion of miRNA associated mRNA are not destabilised and degraded. Rather, they may be protected and display some alternative cellular function. This hypothesis was supported by the divergence of pathways enriched with target genes that are positively correlated with the miRNA compared to the canonical negatively correlated relationship. We suspect that these miRNA-mRNA relationships and the alternative fates of the transcript are important for complex cellular functions, particularly in neurons, and hence warrant further investigation.

## Methods

### Cell culture and modulation of miRNA expression

HeLa, HEK-293 and SH-SY5Y cell lines were maintained as confluent monolayers at 37°C with 5% CO_2_ and 90% humidity in Dulbecco’s Modified Eagle Medium (DMEM) with 10% (vol/vol) foetal bovine serum (Bovagen), 20mM HEPES (4-(2-hydroxyethyl) piperazine-1-ethanesulfonic acid) and 2mM L-glutamine. miRNA expression was modulated in cultured cells *in vitro* using synthetic miRNA or LNA-modified anti-miR oligonucleotides (see Additional file 1: Table S1 for all oligonucleotide sequences). In each case, these oligos were delivered by either transfection or electroporation as described previously [70,71]. HeLa and HEK-293 cells were seeded in 10cm petri dishes at 5 × 10^6^ cells/well, and transfected 24hrs later using Lipofectamine 2000 (Invitrogen) according to manufacturer’s instructions. SH-SY5Y cultures (1 × 10^6^ cells) were subjected to electroporation using Nucleofector Kit V according to the manufacturer’s protocol (Amaxa). All cell lines were transfected with either 100nM siRNA or 250nM LNA-anti-miR oligos, with HEK-293 and HeLa cells compared against their non-transfection controls, and SH-SY5Y cells against an siRNA control targeting the enhanced green fluorescent protein (EGFP).

### Gene expression microarray analysis

Total RNA was extracted from cultured cells 24-hours post-transfection using TRIzol reagent (Invitrogen), and quantitative real-time RT-PCR performed as previously described [72] to validate successful modulation of miRNA expression from the transfection experiments. The extracted RNA was prepared for gene expression analysis using an RNeasy MinElute cleanup kit (Qiagen), followed by biotin-labelling RNA amplification with the TotalPrep RNA amplification kit (Ambion). The labelled RNA was subsequently hybridised to Illumina HumanRef-8 whole-genome expression BeadChips, and scanned using an Illumina BeadArray Reader. RNA concentration was measured using a Quant-iT RiboGreen RNA assay kit and Qubit fluorometer (Invitrogen). All procedures were performed according to manufacturer’s instructions. Data was obtained using Beadstudio v3.2, and analysed using GeneSpring GX 7.3.1.

Default settings in GeneSpring were utilised to execute both per gene and per chip normalisation, as well as to generate gene- and condition-based hierarchical clustering. Genes were excluded from analyses if their expression was below-background in more than half of the samples for each cell line. Gene expression levels in control treatment samples were measured as a reference point for differential gene expression analysis. Genes were considered differentially expressed if changed by more than 1.5-fold in response to modulation of miRNA expression. For the purpose of exploratory analysis, all genes with a p<0.05 (non-corrected) were considered. For pathways analysis of more restricted gene sets consistent through bidirectional modulation of miRNA, we reduced the threshold further to include genes on the basis of fold change alone.

### Bioinformatic analyses

The functional annotation tool of the Database for Annotation, Visualization, and Integrated Discovery (DAVID) bioinformatics resource [73] (http://david.abcc.ncifcrf.gov/) was used to analyse target genes of interest, whether predicted and/or identified from differential gene expression analysis, and used to identify significantly enriched KEGG pathways against a *homo sapiens* background. Venn Diagrams were generated using Venny [74].

miRNA target predictions were downloaded from TargetScan Human Release 5.2, with predicted target genes for miR-181b and miR-107 categorised by cross-species conservation and seed-region composition before being correlated against the observed gene expression changes subsequent to miRNA modulation. Seed region composition was defined as follows: “8mer” have an ‘A’ at position 1 and perfect complimentary from positions 2–8 of the mature miRNA; “7mer-m8” have perfect complementarity from positions 2–8 of the mature miRNA; and “7mer-1A” have an ‘A’ at position 1 and perfect complementarity to positions 2–7 of the mature miRNA. A ‘true positive’ was defined as a predicted target gene that was differentially expressed in the direction for canonical miRNA function; ‘true negatives’ were those genes not predicted as miRNA targets and not differentially expressed; a ‘false positive’ was a gene predicted to be a miRNA target, but not differentially expressed with miRNA modulation; and ‘false negatives’ were those genes not predicted to be miRNA targets but differentially expressed in the direction corresponding to canonical miRNA function. Targets from miRGen [75] were also included where specified. Subsequent analysis of non-canonical miRNA function was carried out as described above. Canonical miRNA function was defined with respect to the conventional expectation of an inverse relationship between miRNA and mRNA expression, whereas non-canonical miRNA function was defined as the positive correlation observed between miRNA and mRNA expression levels.

The accuracy of the Targetscan algorithm to predict observed biological changes was determined by the sum of all ‘true positive’ and ‘true negative’ observations as a percentage of all ‘true positive’, ‘true negative’, ‘false positive’, and ‘false negative’ observations. The sensitivity was determined by calculating the number of ‘true positives’ divided by the number of ‘true positives’ and ‘false negatives’, thus giving an indication of the proportion of observed changes that were predicted correctly by the algorithm. This is represented as a value between zero and one, with a high sensitivity indicating a low ‘false negative’ rate (FNR); the FNR (Type II error) is calculated as [1-sensitivity]. Specificity was calculated as the number of ‘true negatives’ divided by the sum of ‘true negatives’ and ‘false positives’. This is represented as a value between zero and one, with a high specificity indicating a low ‘false positive’ rate (FPR); the FPR (Type I error) is calculated as [1-specificity]. Statistical analyses were performed using GraphPad Prism 5, where repeated measures ANOVAs (rmANOVAs) and Student’s t-tests (paired, two-tailed) were performed to investigate differences between various parameters, whilst correlation was used to investigate similarities between parameters of canonical and non-canonical responses.

The TRANSFAC [76] function of GATHER [77] (http://gather.genome.duke.edu/) was used to identify enrichment of specific transcription factor signatures within differentially expressed genes. A Bayes Factor of 6, which in each case corresponded to a p-value <0.0001, was used as a threshold for statistical significance. AU-rich elements were identified using the Organism function of the ARE database (http://brp.kfshrc.edu.sa/AredOrg/) [78]. Potential MREs in genes of interest were identified using miRanda v1.0 software [79], with 3′-UTR information obtained using AceView [80]. Genes associated with schizophrenia were selected from the Schizophrenia Gene Database Index (http://www.schizophreniaforum.org/res/sczgene/dbindex.asp).

### miRNA target-gene reporter assays

Putative miR-181b MREs containing synthetic sequences were cloned into *Spe* I and *Hind* III sites in the multiple cloning region downstream of the firefly luciferase gene in pMIR-REPORT (Ambion) backbone as described [27,28,71]. To achieve this, 4μg pMIR-REPORT was incubated for two hours at 37°C with 2U each Spe I and Hind III, 10U of T4 DNA ligase, and 10μM of double-stranded DNA oligonucleotide of potential miR-181b recognition element.

Validation of putative MREs was performed using the dual luciferase reporter gene assay (Promega) in a 96-well format, with 4x10^4^ cells seeded per well. Lipofectamine 2000 was used to transfect SH-SY5Y cells 24 hours post-seeding, with 1.33nM synthetic miRNA co-transfected along with 2ng recombinant pMIR-REPORT firefly luciferase reporter gene construct, and 12ng pRL-TK renilla luciferase vector as an internal transfection control. The response of recombinant 3′-UTR motifs to miR-181b was normalised with respect to a mutant miR-181b control (miR-181b_mut, designed from the miR-181b backbone with mutations introduced at positions 3, 5, and 7; see Additional file 1: Table S1 for oligonucleotide sequences). For validation of miRNA interaction with E2F1, 50ng E2F1 3′-UTR reporter (Switchgear Genomics) was co-transfected into HEK-293 cells with 15ng pRL-TK and either 30nM miRNA or 100nM anti-miR inhibitor. For each miRNA and anti-miR inhibitor, reporter gene expression was normalised against miR-26b and anti-miR-16_scr4 oligonucleotide controls respectively, designed and selected due to their minimal predicted binding across the entirety of the E2F1 3′-UTR. For each condition, experiments were performed n=4 times, each with internal triplicates (to generate mean data values), and a paired t-test performed to compare responses between treatment and control oligos.

## Abbreviations

miRNA: microRNA; RISC: RNA induced silencing complex; E2F1: E2F transcription factor 1; MRE: miRNA recognition element; 3′: UTR: 3′ untranslated region; ceRNA: Competing endogenous RNA; LNA: Locked-nucleic acid; rmANOVA: Repeated measures analysis of variance; FPR: False positive discovery rate; FNR: False negative discovery rate; P_CT_: Probability of conserved targeting; ARE: AU-rich element.

## Competing interests

The authors declare that they have no competing interests.

## Authors' contributions

APC contributed to the design of this study, developed the experimental techniques and carried out this study, analysed and interpreted the data, and wrote the manuscript. NT contributed to the study design. PAT provided technical assistance and contributed to the manuscript preparation. MJC conceived the initial study design, participated in its design and implementation, and co-wrote the manuscript. All authors read and approved the final manuscript.

## Supplementary Material

Additional file 1 **Supplementary Tables.** Contains supplementary tables S1–S10. This includes sequence information for all oligonucleotides used in this study, along with enriched KEGG pathways, p-values, and contributing genes for experimental conditions in each cell type. Click here for file

Additional file 2 **Figure S1.** Conservation scores for modulated predicted miR-181b targets, as predicted using Targetscan. PCT: Probability of conserved targeting; the lower the probabilistic value, the poorer the conservation of the predicted binding site across multiple species. Bidirectional 2+ indicates genes modulated by both miR-181b over-expression and inhibition across two or more cell models. Click here for file

Additional file 3 **Figure S2.** Bioinformatic evidence for a role of E2F1 transcription factor in contributing to miRNA-associated expression profiles. Panel A graphically represents the Transcription factor association of canonically modulated genes subsequent miR-181b over-expression or inhibition in HEK-293, HeLa, and SH-SY5Y cell models. The TRANSFAC function of GATHER was used to identify significantly enriched transcription factor signatures within modulated genes. A Bayes factor of 6 was used for threshold significance, which in each case corresponded to p<0.0001. Panel B illustrates predicted binding sites for schizophrenia-associated miR-181b, miR-107, and miR-20a in the 3′-UTR of E2F1, as well as the AU-rich element in this 3′-UTR. These predicted binding sites were identified using the miRanda shell algorithm; only sites with threshold scores greater than 120 are shown in this figure. Click here for file

Additional file 4 **Figure S3.** Distribution of predicted miRNA and E2F1 function for modulated mRNA. Panel A shows the proportion of modulated genes that can be attributed to predicted miR-181b and E2F1 function across individual and multiple cell models; whilst Panel B shows this for miR-107 predicted function. In both panels data is presented for canonical (negative miRNA-mRNA correlation) and non-canonical (positive miRNA-mRNA correlation) patterns of correlation. Differentially expressed genes are classified as being predicted in preferential fashion by: Targetscan conserved predicted miR-181b target; Targetscan non-conserved predicted miR-181b target, with PCT (probability of conservation) scores <0.1; Targetscan non-conserved predicted miR-181b target, with PCT scores >0.1; not predicted as a miR-181b target by Targetscan, but predicted by the miRGen algorithm; predicted as containing E2F1 recognition signatures using the TRANSFAC algorithm. Click here for file

Additional file 5 **Figure S4.** Bidirectional modulation of miR-107 expression. Panel A shows enriched KEGG pathways from predicted miR-107 target genes. Panel B shows the modulation of miR-107 expression levels in HEK-293 and HeLa cell types, indicative of miR-107 over-expression and inhibition. Panel C illustrates enriched KEGG pathways from modulated mRNA subsequent to miR-107 over-expression in HEK-293 and HeLa cell models. Panel D illustrates enriched KEGG pathways from modulated mRNA subsequent to miR-107 inhibition in HEK-293 and HeLa cell models. Panel E shows Venn diagrams and subsequent KEGG pathways analyses for the intersection of bidirectionally modulated genes in each cell type; and for the union of modulated genes across multiple cell models. The intersection of bidirectionally-modulated genes identifies genes modulated by both miR-107 over-expression and inhibition in each cell type. Genes modulated by either over-expression or inhibition were considered for the union of modulated genes across multiple cell types. The subsequent KEGG pathways analyses on these genes of interest revealed significantly enriched pathways, as evident in the bottom half of this panel. R-M: Receptor-mediated. RI: receptor interaction. ECM: Extracellular matrix. ARVC: Arrhythmogenic right ventricular cardiomyopathy. Click here for file
