# Supplementary material for: Alternative mRNA fates identified in microRNA-associated transcriptome analysis
Source: BMC Genomics. 2012 Oct 19;13:561. doi: 10.1186/1471-2164-13-561 (PMC3505728; doi:10.1186/1471-2164-13-561)
Supplement: Additional file 1 — Supplementary Tables. Contains supplementary tables S1–S10. This includes sequence information for all oligonucleotides used in this study, along with enriched KEGG pathways, p-values, and contributing genes for experimental conditions in each cell type. [file 1471-2164-13-561-S1.docx]

# Supplementary Tables

## Table 1. Oligonucleotide sequences.

| **Type** | **Name** | **Sequence** | **Target** |
| --- | --- | --- | --- |
| miRNA^a^ | miR-181b^+^ | AACAUUCAUUGCUGUCGGUGGG | miR-181b |
|  | miR-181b^–^ | CACCGACAGCAAUGAAUGUUUU | miR-181b |
|  | miR-181b_mut^+^ | AAAAAUAAUUGCUGUCGGUGGG | miRNA transfection control |
|  | miR-181b_mut^–^ | CACCGACAGCAAUUAUUUUUUU | miRNA transfection control |
|  | miR-107^+^ | AGCAGCAUUGUACAGGGCUAUCA | miR-107 |
|  | miR-107^–^ | AUAGCCCUGUACAAUGCUGUAUU | miR-107 |
|  | miR-107_mut^+^ | UCCACCAAUGUACAGGGCUAUCA | miRNA transfection control |
|  | miR-107_mut^–^ | AUAGCCCUGUACAUUGGUGAAUU | miRNA transfection control |
|  | miR-20a^+^ | UAAAGUGCUUAUAGUGCAGGUAG | miR-20a |
|  | miR-20a^–^ | ACCUGCACUAUAAGCACUUUAUU | miR-20a |
|  | miR-26b^+^ | UUCAAGUAAUUCAGGAUAGGU | miRNA transfection control |
|  | miR-26b^–^ | CUAUCCUGAAUUACUUGAAUU | miRNA transfection control |
| siRNA | si-EGFP^+^ | CGGCAAGCUGACCCUGAAGUU | miRNA transfection control |
|  | si-EGFP^–^ | CUUCAGGGUCAGCUUGCCGUU | miRNA transfection control |
| anti-miR^b^ | Anti-miR-181b | C^CCA^CCG^ACA^GCA^ATG^AAT^GT | miR-181b |
|  | Anti-miR-181b_mut | C^CCA^GCG^ACA^CCA^ATG^AAT^CT | Anti-miR transfection control |
|  | Anti-miR-107 | T^GAT^AGC^CCT^GTA^CAA^TGC^TG | miR-181b |
|  | Anti-miR-107_mut | T^GAT^AGC^CCT^GTA^CAT^TGG^TG | Anti-miR transfection control |
|  | Anti-miR-20a | CTA^CCT^GCA^CTA^TAA^GCA^CTT^TA | miR-20a |
|  | Anti-miR-16_scr4 | C^GCC^AAT^ATT^TAC^GTG^GTG^GAT | Anti-miR transfection control |
| Primers^c^ | U6_F | CGGCAGCACATATACTAAAATTGG | U6 snRNA |
|  | U6_R | GCCATGCTAATCTTCTCTGTATC | U6 snRNA |
|  | 181b_F | TTTCTAACATTCATTGCT | miR-181b |
|  | 181b_R | CAACCTTCTCCCACCGAC | miR-181b |
| Casettes^d^ | BIK^T^ | CTAGAATTCCGGGAATAGATTCCGAGGAGCAGGAGTGCTCAATAAA | BIK |
|  | BIK^B^ | AGCTTTTATTGAGCACTCCTGCTCCTCGGAATCTATTCCCGGAATT | BIK |
|  | CHRNA2^T^ | CTAGAATTCCGGTCTTATCACTGGCTGGAGAGCAACGTGGATGCCGAGGAG | CHRNA2 |
|  | CHRNA2^B^ | AGCTCTCCTCGGCATCCACGTTGCTCTCCAGCCAGTGATAAGACCGGAATT | CHRNA2 |
|  | DISC1 ^T^ | CTAGAATTCCGGTTCTATTCTAGTTCATTAAAAGTGAATGTTGGTCTT | DISC1 |
|  | DISC1 ^B^ | AGCTAAGACCAACATTCACTTTTAATGAACTAGAATAGAACCGGAATT | DISC1 |
|  | ENKUR_1^T^ | CTAGAATTCCGGAAAAATCCTTAATGAATAAAGTAATGGATCGTACCCAAA | ENKUR/c10orf63 |
|  | ENKUR_1^B^ | AGCTTTTGGGTACGATCCATTACTTTATTCATTAAGGATTTTTCCGGAATT | ENKUR/c10orf63 |
|  | ENKUR_2^T^ | CTAGAATTCCGGCGTTTGCATCGCTAAGTAAGCAACTTAAGTTGCTTTAACAA | ENKUR/c10orf63 |
|  | ENKUR_2^B^ | AGCTTTGTTAAAGCAACTTAAGTTGCTTACTTAGCGATGCAAACGCCGGAATT | ENKUR/c10orf63 |
|  | FGA_1^T^ | CTAGAATTCCGGAACTCTTCCACTAGACGTTGTAATGCACACTTATTTT | FGA |
|  | FGA_1^B^ | AGCTAAAATAAGTGTGCATTACAACGTCTAGTGGAAGAGTTCCGGAATT | FGA |
|  | FGA_2^T^ | CTAGAATTCCGGATGGCTTTTGATCCAGCAAAGAATGGATGGATCACTGAA | FGA |
|  | FGA_2^B^ | AGCTTTCAGTGATCCATCCATTCTTTGCTGGATCAAAAGCCATCCGGAATT | FGA |
|  | GPR78 ^T^ | CTAGAATTCCGGACACTAGACGCCCAAAGCAGGATGTGTCTTTTGGG | GPR78 |
|  | GPR78 ^B^ | AGCTCCCAAAAGACACATCCTGCTTTGGGCGTCTAGTGTCCGGAATT | GPR78 |
|  | KCNMB2 ^T^ | CTAGGAGTGTCATTACCTGTGAGCTGACTGAATGTTGGTAGG | KCNMB2 |
|  | KCNMB2 ^B^ | AGCTCCTACCAACATTCAGTCAGCTCACAGGTAATGACACTC | KCNMB2 |
|  | MTMR1^T^ | CTAGTGTAATCCCCTGGCTGACTAGGACTGTTAAACAT | MTMR1 |
|  | MTMR1^B^ | AGCTATGTTTAACAGTCCTAGTCAGCCAGGGGATTACA | MTMR1 |
|  | MMP14^T^ | CTAGCGCTCCCCCACCCAGCCCACCCATTGAAGTCTCCTTGG | MMP14 |
|  | MMP14^B^ | AGCTCCAAGGAGACTTCAATGGGTGGGCTGGGTGGGGGAGCG | MMP14 |
|  | NR6A1_1 ^T^ | CTAGAATTCCGGATTTATTTCACGACAGAGTTGAATGTATGGCCTT | NR6A1 |
|  | NR6A1_1 ^B^ | AGCTAAGGCCATACATTCAACTCTGTCGTGAAATAAATCCGGAATT | NR6A1 |
|  | NR6A1_2 ^T^ | CTAGAATTCCGGTGAAAAACCAGCTGAGCAGAATGCCATGTTCTGAAG | NR6A1 |
|  | NR6A1_2 ^B^ | AGCTCTTCAGAACATGGCATTCTGCTCAGCTGGTTTTTCACCGGAATT | NR6A1 |
|  | SLC22A7 ^T^ | CTAGCCGAGGCACCCTGCAGGGCAATGCATGTCATCCCA | SLC22A7 |
|  | SLC22A7 ^B^ | AGCTTGGGATGACATGCATTGCCCTGCAGGGTGCCTCGG | SLC22A7 |
| ^a^ Synthetic miRNA were designed to mimic the endogenous miRNA, with ‘+’ indicating the mature miRNA strand, and ‘–’ indicating the passenger strand. ^b^ Anti-miR oligonucleotides were designed complementary to the mature miRNA, with LNA-modified nucleotides preceded by a ‘^’ symbol. ^c^ The direction of the forward ‘F’ and reverse ‘R’ primers are in respect to the target sequence. ^d^ SpeI/HindIII cassettes containing putative miRNA recognition elements were used to generate recombinant luciferase reporter-gene constructs, with ‘T’ indicating the top strand (containing the putative binding site), and ‘B’ indicating the bottom strand. | | | |

## Table 2. Enriched KEGG pathways of predicted miR-181b target genes.

| **Term** | **Count** | **PValue** | **Genes** |
| --- | --- | --- | --- |
| TGF-beta signalling pathway | 20 | 0.002311 | ACVR2A, SMAD9, ID4, SMAD6, NOG, DCN, E2F5, CREBBP, ACVRL1, SMAD7, PPP2R2C, RPS6KB1, IFNG, THBS4, ACVR2B, INHBA, LEFTY2, TGFBR1, EP300, BMPR2. |
| Prostate cancer | 19 | 0.003762 | PIK3R3, PDGFA, LEF1, BCL2, AKT3, CDKN1B, CREB3L4, KRAS, INSRR, CREB1, CREBBP, FGFR2, HSP90B1, ERBB2, PDGFRA, CHUK, MAP2K1, EP300, CREB3. |
| Neurodegenerative Diseases | 11 | 0.006086 | SETX, GFAP, VAPB, NEFH, HSPA5, BCL2, CASP1, ALS2, EP300, LRRK2, CREBBP. |
| Melanogenesis | 20 | 0.006242 | KIT, POMC, LEF1, FZD4, PRKACG, CREB3L4, KRAS, CREB1, CREBBP, ADCY9, ADCY1, WNT11, MITF, MAP2K1, CAMK2G, GNAO1, GNAI1, EP300, CREB3, DCT. |
| Long-term potentiation | 15 | 0.007 | PPP3R1, RAP1B, GRIA2, GRM1, PRKACG, KRAS, GRM5, RPS6KA3, CREBBP, ADCY1, PPP3CA, MAP2K1, CAMK2G, EP300, PPP1CB. |
| T cell receptor signalling pathway | 19 | 0.008742 | VAV3, PIK3R3, IL2, PPP3R1, CBLB, AKT3, KRAS, CD4, IFNG, PAK7, PPP3CA, FOS, PLCG1, NFAT5, CHUK, MAP3K8, FYN, CARD11, PAK4. |
| Axon guidance | 24 | 0.010615 | PPP3R1, SEMA4G, SEMA7A, EPHA7, NTN4, EPHA4, KRAS, SRGAP1, CFL2, UNC5B, NRP1, UNC5A, PAK7, PPP3CA, SEMA4C, NFAT5, FYN, SLIT2, DPYSL2, NGEF, SEMA3B, PAK4, GNAI1, SLIT1. |
| MAPK signalling pathway | 39 | 0.021684 | MAP3K7IP2, RRAS, PDGFA, IL1B, MAP3K6, PRKACG, KRAS, MAP3K3, RPS6KA3, DUSP5, MKNK2, FGFR2, MAP3K5, PDGFRA, CHUK, PTPRR, CACNB2, GNA12, CACNA2D2, PPP3R1, IL1A, RAP1B, AKT3, MAP4K4, NLK, PLA2G4A, ARRB2, RASGRP4, MAP3K10, PPP3CA, FOS, MAP3K8, MAP2K1, TGFBR1, PPM1B, DUSP6, FGFR3, MAP3K12, DUSP10. |
| Dorso-ventral axis formation | 8 | 0.023637 | NOTCH4, CPEB1, ERBB2, ETV6, SPIRE1, MAP2K1, KRAS, ETS1. |
| Circadian rhythm | 5 | 0.041049 | BHLHB2, CSNK1D, CLOCK, BHLHB3, PER2. |
| hsa05221:Acute myeloid leukemia | 11 | 0.060617964 | KIT, STAT3, RPS6KB1, RUNX1T1, PIK3R3, LEF1, CHUK, MAP2K1, AKT3, RUNX1, KRAS, |
| hsa04810:Regulation of actin cytoskeleton | 31 | 0.064570576 | VAV3, RRAS, ARPC1A, PDGFA, IQGAP2, KRAS, ITGA4, ARPC1B, CFL2, FGFR2, MYH10, APC, PDGFRA, BAIAP2, WASL, ARPC5L, GNA12, PPP1CB, PIK3R3, SSH1, BDKRB1, ITGA3, ITGB8, IQGAP3, PAK7, MAP2K1, PIP5K3, PPP1R12B, PAK4, FGFR3, ARHGEF7, |
| hsa04540:Gap junction | 16 | 0.07349087 | PRKG1, GJA1, PDGFA, GRM1, PRKACG, KRAS, GRM5, HTR2C, ADCY1, ADCY9, TUBB6, CSNK1D, PDGFRA, MAP2K1, GNAI1, TUBAL3, |
| hsa04612:Antigen processing and presentation | 14 | 0.076313946 | NFYC, KIR2DS2, KIR2DS4, KIR3DL2, KIR2DL1, HSPA5, KIR3DL3, IFNA17, CREB1, KIR2DS1, CD4, KIR2DS5, HSP90AB1, KIR2DS3, |
| hsa05211:Renal cell carcinoma | 12 | 0.092462862 | EPAS1, PIK3R3, PAK7, MAP2K1, RAP1B, AKT3, PAK4, KRAS, ETS1, EP300, CREBBP, ARNT2, |

## Table 3A. Enriched KEGG pathways of downregulated genes with miR-181b over-expression in HEK-293 cells.

| **Term** | **Count** | **PValue** | **Genes** |
| --- | --- | --- | --- |
| Neuroactive ligand-receptor interaction | 73 | 6.71E-10 | P2RY6, BRS3, GRIN2D, GPR35, PRL, GCGR, MTNR1A, GH1, DRD1, HTR2A, GABRR1, P2RX1, P2RY8, P2RY1, MC3R, P2RY10, ADRA2A, P2RX2, EDG1, DRD2, GABRA5, GRIN2C, AVPR2, P2RY2, GABRG2, GRPR, HTR5A, GRIN2B, CRHR2, NPY1R, GZMA, ADRB3, GRIK2, HRH2, GH2, ADORA3, CHRM2, FPRL2, LEPR, HTR1D, HRH3, GABRB3, AGTRL1, NPY5R, EDNRA, GRM6, HRH4, HRH1, SSTR5, GABRP, P2RY5, SSTR1, LTB4R2, GRIN1, GRIA1, HTR1F, GRIK3, GALR3, TSHB, EDNRB, GABRE, PRLR, GABRA1, GRIA3, FSHB, HTR7, PPYR1, F2RL2, GLP2R, PTGER1, TRPV1, CYSLTR2, TACR1. |
| Cytokine-cytokine receptor interaction | 60 | 4.46E-05 | IL11, CCR5, IFNW1, CSF3, PRL, XCL2, TNFRSF14, PF4, CCL11, EGFR, GH1, CCL18, CNTFR, TNFSF14, IL6ST, IL22RA1, CCR1, CCL24, IL17RB, HGF, TNFRSF10C, INHBE, CCR4, TNFRSF8, CCL21, TNFRSF18, CXCL12, IL2RA, IFNAR1, GH2, CCL28, IL20RA, LEPR, CCR3, IL10RA, CXCR3, TNFSF12, MPL, IL9R, OSM, NGFR, IL3, MET, IL28B, IL5, TGFB1, CTF1, KITLG, TNFSF10, CCL23, PDGFRB, PRLR, IL18RAP, IL1R2, IL5RA, CCL26, FLT3LG, TNFRSF1B, IL4, XCR1. |
| Hematopoietic cell lineage | 25 | 4.42E-04 | CD5, DNTT, IL11, GP9, CD8A, ITGA4, CSF3, IL9R, IL3, CD7, IL5, CD24, FCGR1A, KITLG, IL1R2, CD1E, IL5RA, GYPE, CD33, CR1, MME, FLT3LG, MS4A1, IL4, IL2RA. |
| Cell adhesion molecules (CAMs) | 30 | 0.005194 | CD226, ITGAL, CNTN1, NRXN1, CTLA4, CD6, CLDN2, ITGB7, CD8A, CLDN23, ITGA4, CLDN4, CNTN2, CDH3, HLA-DQB2, HLA-DOA, PTPRC, NFASC, CLDN7, JAM2, ITGB1, SELPLG, CDH15, CDH5, SELE, F11R, SDC2, ESAM, SPN, L1CAM. |
| Calcium signalling pathway | 38 | 0.005344 | BST1, CHRM2, GRIN2D, PLCD4, PRKACG, EDNRA, PLCD1, HRH1, PPP3R2, LTB4R2, GRIN1, TNNC2, EGFR, DRD1, HTR2A, SLC8A3, EDNRB, PLCE1, P2RX1, RYR2, ATP2B1, P2RX2, PDGFRB, ADCY9, GRIN2C, PLCB1, HTR7, CACNA1G, GRPR, HTR5A, PTK2B, GNA14, ADRB3, PTGER1, CYSLTR2, TACR1, CACNA1D, HRH2. |
| Melanoma | 17 | 0.027086 | PIK3CA, CDKN1A, FGF5, IGF1, CDKN2A, AKT3, FGF19, PIK3R1, PDGFRB, HGF, PDGFD, MDM2, FGF2, FGF22, EGFR, MET, FGF10. |
| Jak-STAT signalling pathway | 31 | 0.030947 | IFNAR1, GH2, IL20RA, IL11, LEPR, SPRY3, IL10RA, MPL, IFNW1, CSF3, PRL, IL9R, OSM, IL3, GH1, IL28B, IL5, PIK3CA, CNTFR, IL6ST, SPRY1, CTF1, AKT3, STAM2, IL22RA1, PIK3R1, PRLR, IL5RA, STAT5A, IL4, IL2RA. |
| Arachidonic acid metabolism | 14 | 0.038036 | PLA2G10, CYP2E1, PLA2G4A, PTGDS, PLA2G2A, TBXAS1, PLA2G3, CYP2C8, CYP4A11, CYP4F2, PTGIS, PLA2G2E, CYP2B6, GPX5. |
| Linoleic acid metabolism | 9 | 0.070393435 | PLA2G10, PLA2G3, AKR1B10, CYP2C8, CYP2E1, PLA2G4A, PLA2G2A, PLA2G2E, CYP3A5, |
| Leukocyte transendothelial migration | 23 | 0.090646291 | PIK3CA, CYBB, ITGAL, CLDN7, JAM2, PIK3R1, CLDN2, RAC2, ITGB1, CLDN23, ITGA4, CTNNA3, ACTN3, NCF2, CLDN4, CDH5, RAPGEF3, RAC1, PTK2B, CXCL12, F11R, ROCK1, ESAM, |

## Table 3B. Enriched KEGG pathways of upregulated genes with miR-181b inhibition in HEK-293 cells.

| **Term** | **Count** | **PValue** | **Genes** |
| --- | --- | --- | --- |
| Melanoma | 21 | 0.004714 | AKT2, BAD, FGF21, MAP2K2, FGF8, FGF3, CDKN2A, TP53, FGF12, AKT3, PIK3R1, PIK3R5, RB1, FGF20, PIK3CB, FGF2, CDH1, FGF22, EGFR, FGF18, FGF13. |
| MAPK signalling pathway | 57 | 0.009585 | RAP1A, FGF21, IL1B, FGF3, FGF8, FGF12, FGF20, CACNG1, EGFR, CACNB1, PPM1A, RASGRF2, AKT2, MAP2K2, MAP3K4, RRAS2, AKT3, GNG12, RASGRF1, MAP4K3, CACNG5, FOS, MAP2K3, CACNA1G, MAPT, FGF22, PLA2G2E, DUSP10, PLA2G10, MAP2K6, RASA2, CACNA1H, IL1R1, SOS1, CACNG2, FGF2, PTPRR, MAP3K7IP1, FGF13, GNA12, NTRK1, NR4A1, MAPK10, CACNG7, TP53, DUSP4, FLNA, CACNA1E, CACNB4, ACVR1B, CACNG3, DUSP7, FGF18, PPM1B, MAPK8IP2, CDC25B, CACNA1D. |
| Endometrial cancer | 16 | 0.009701 | ILK, AKT2, AXIN1, PDPK1, CTNNB1, BAD, MAP2K2, TP53, AKT3, PIK3R1, PIK3R5, SOS1, PIK3CB, CDH1, EGFR, TCF7. |
| Acute myeloid leukemia | 17 | 0.010538 | AKT2, RUNX1T1, CEBPA, BAD, PML, MAP2K2, RPS6KB2, JUP, AKT3, RUNX1, PIK3R1, RELA, PIK3R5, SOS1, PIK3CB, RARA, TCF7. |
| Renin-angiotensin system | 8 | 0.011985 | CTSG, ACE, NLN, MME, LNPEP, ANPEP, MAS1, CPA3. |
| Non-small cell lung cancer | 16 | 0.014151 | AKT2, RASSF1, PDPK1, BAD, MAP2K2, CDKN2A, TP53, AKT3, PIK3R1, PIK3R5, RB1, SOS1, PIK3CB, EGFR, RASSF5, RXRG. |
| Neuroactive ligand-receptor interaction | 55 | 0.01517 | GABRB1, P2RY6, P2RX3, UTS2R, F2RL3, GPR35, ADRA1A, GLP1R, TACR2, ADRB1, GH1, DRD1, GLRA2, HTR2A, NPBWR1, GALR1, CHRM5, P2RY13, EDG1, GABRA3, TSHR, GABRA5, GABRA4, NR3C1, HCRTR1, PTGER3, HRH2, VIPR2, ADORA3, CHRM2, LEPR, HRH4, GABRQ, GABRA6, CALCRL, GRIK1, LTB4R2, GRIA1, OPRM1, GPR50, HTR1F, GALR3, LEP, EDNRB, EDG2, CTSG, GRIK4, OPRL1, ADRA2C, MC2R, NPFFR2, CNR2, MAS1, CYSLTR2, EDG7. |
| Thyroid cancer | 10 | 0.02096 | CTNNB1, MAP2K2, TP53, PPARG, PAX8, CDH1, RET, TCF7, RXRG, NTRK1. |
| Type I diabetes mellitus | 12 | 0.038887 | HSPD1, IL12B, IL1B, HLA-A, PRF1, INS, HLA-F, HLA-DOB, HLA-DQB2, HLA-DQA2, HLA-C, PTPRN2. |
| Hematopoietic cell lineage | 21 | 0.046068 | CD34, CD36, IL11RA, IL1B, ANPEP, GP9, KITLG, ITGA6, GYPE, CD4, IL1R1, CD2, MME, IL3, FCER2, ITGA2, IL2RA, CD44, CD3G, TPO, IL5. |
| Prostate cancer | 21 | 0.057048428 | AKT2, CTNNB1, PDPK1, BAD, MAP2K2, INS, TP53, AKT3, PIK3R1, CDKN1B, RELA, INSRR, PIK3R5, RB1, SOS1, PIK3CB, CREB3L1, EGFR, AR, TCF7, CREB3L3, |
| Regulation of actin cytoskeleton | 44 | 0.064565875 | ACTN2, CHRM2, FGF21, FGF8, FGF3, MLCK, INS, FGF12, ARPC2, ITGA6, CFL2, ITGB2, PIK3R5, GRLF1, FGF20, SOS1, PPP1CA, BAIAP2, PIK3CB, FGF2, EGFR, ITGA2, GNA12, FGF13, RDX, PFN2, CHRM5, SSH1, MAP2K2, RRAS2, GNG12, PIK3R1, ITGB1, TIAM2, ACTB, GSN, PIP5K1B, ITGAX, FGF22, PFN1, PPP1R12B, FGF18, ARHGEF1, MYLK, |
| Chronic myeloid leukemia | 18 | 0.075718046 | AKT2, BAD, SHC4, MAP2K2, CDKN2A, CBLC, TP53, AKT3, RUNX1, CDKN1B, PIK3R1, RELA, ACVR1B, PIK3R5, RB1, SOS1, PIK3CB, SHC1, |
| Jak-STAT signaling pathway | 32 | 0.089780418 | IL11RA, IL12B, IL28A, CBLC, LEPR, SPRY3, IL10RA, PTPN6, PIK3R5, IL10RB, JAK1, IL12RB1, SOS1, IL3, PIK3CB, GH1, LEP, IL5, AKT2, IL21R, IFNA21, IL19, AKT3, STAM2, PIK3R1, JAK3, TSLP, IFNK, SOCS5, SPRY2, IL2RA, TPO, |
| Small cell lung cancer | 20 | 0.095430301 | AKT2, LAMA2, NOS2A, CDKN2B, TP53, AKT3, BIRC2, CDKN1B, PIK3R1, ITGA6, RELA, ITGB1, PIK3R5, RB1, TRAF4, PIK3CB, COL4A6, ITGA2, RXRG, LAMA3, |
| Type II diabetes mellitus | 12 | 0.096073121 | PRKCZ, IRS2, PKM2, MAPK10, CACNA1G, PIK3CB, INS, PIK3R1, CACNA1E, PRKCD, CACNA1D, PIK3R5, |
| Ubiquitin mediated proteolysis | 28 | 0.097691033 | UBE2A, CBLC, UBE2G1, CDC34, MGRN1, DET1, CUL5, BRCA1, KEAP1, CUL4B, RCHY1, SIAH1, PML, TCEB1, HERC3, BIRC2, NHLRC1, TRIM37, UBE2D3, UBE2W, UBE2I, PARK2, WWP2, RNF7, PPIL2, FANCL, UBE2C, NEDD4, |

## Table 4A. Enriched KEGG pathways of downregulated genes with miR-181b over-expression in HeLa cells.

| **Term** | **Count** | **PValue** | **Genes** |
| --- | --- | --- | --- |
| Cytokine-cytokine receptor interaction | 71 | 1.56E-07 | CXCL2, TNFSF8, IL23A, CCR5, TNFRSF19, IFNW1, CSF3, PRL, TNFRSF14, PDGFB, CCL11, GH1, EPO, CXCL5, CCL18, CXCL3, CNTFR, CCL14, TNFSF14, IL6ST, TNFRSF10B, IL17RB, CCR7, IL2RB, IL7, IFNA1, CXCL12, IL20, IFNB1, IL12B, CXCL14, IL20RA, TNFSF13B, IL3RA, CCL13, CXCR6, OSM, IL1RAP, IL12RB1, IL29, IL3, CCL17, TNFRSF9, IFNA10, TNFRSF4, TNFRSF11A, TNFRSF10D, IL21R, CSF2RB, TNFRSF6B, IL24, CCL23, ACVR1B, IL8RB, IL1R2, IFNA5, CCR6, IFNG, CXCL1, TGFBR2, IL8, PPBP, INHBA, TNFRSF1B, TNFSF11, CXCL10, KDR, CCL25, CCR9, TGFB2, CXCL13. |
| Neuroactive ligand-receptor interaction | 56 | 0.002788 | P2RX3, F2RL3, GCGR, PRL, GLP1R, PRSS1, PTHR1, ADRB1, GH1, GALR1, GABRR1, HTR1A, GNRHR, FSHR, MC3R, GRIN3A, P2RY10, GABRA3, DRD2, GABRA5, P2RY2, AVPR2, GABRG2, GRPR, HCRTR1, PTGER3, THRA, MC5R, ADORA3, ADRA2B, HTR1E, GABRB2, NPY5R, AGTRL1, EDNRA, CALCRL, GRIK1, SSTR5, GRIN1, HTR1F, LHCGR, GRIK4, GABRE, C3AR1, SSTR4, P2RX7, GABRA1, GPR83, PPYR1, GIPR, DRD3, P2RY4, PTGER1, EDG7, TACR1, CHRM1. |
| Jak-STAT signalling pathway | 36 | 0.005901 | IFNB1, IL12B, IL20RA, IL23A, CBLC, PTPN6, SOCS1, IL3RA, IFNW1, CSF3, PRL, OSM, IL12RB1, IL29, IL3, GH1, IFNA10, EPO, CNTFR, CISH, CSF2RB, IL6ST, IL21R, SPRY1, STAM2, PIK3CG, IL24, IFNA5, IL2RB, IFNG, IL7, STAT4, JAK3, SOCS5, IFNA1, IL20. |
| Taste transduction | 16 | 0.011956 | GNG3, TAS2R50, TAS2R16, PLCB2, PRKACG, ADCY4, SCNN1B, TAS2R4, ACCN1, TAS2R10, TAS2R8, CACNA1A, TAS2R9, TAS2R45, TAS2R44, TAS2R3. |
| MAPK signalling pathway | 53 | 0.016719 | PPP5C, FGF5, FGF12, PRKACG, FGFR2, MAP4K1, CACNA2D1, MAPK11, PDGFB, FGF14, CACNB1, MAP3K13, CACNA1S, MAP3K4, PTPN7, ARRB2, CACNG5, CACNA2D3, MAP2K3, MOS, MAPT, FGF22, CACNB3, CACNA1I, MAP3K7IP2, DDIT3, CACNA1H, MAP3K7IP1, CACNA1A, MAPK14, GNA12, NTRK1, PLA2G1B, TRAF6, NR4A1, CACNG7, MAPKAPK2, MAP4K4, DUSP4, FGF19, JUN, CACNA1E, ACVR1B, IL1R2, DUSP16, TGFBR2, RASGRP2, CACNA1C, BDNF, FGF18, FGF9, TGFB2, MAPK8IP2. |
| Hedgehog signalling pathway | 16 | 0.019699 | DHH, WNT2B, WNT16, PRKACG, WNT7A, WNT6, WNT8B, SHH, CSNK1G1, BMP8A, WNT5B, WNT1, BMP8B, RAB23, WNT7B, WNT4. |
| ECM-receptor interaction | 22 | 0.019988 | LAMA2, ITGA10, SV2C, FNDC4, ITGA6, ITGA11, ITGB7, ITGB1, COL4A4, CHAD, ITGB3, COL11A1, GP5, RELN, THBS4, SV2A, SPP1, COL2A1, COL1A1, FNDC5, COL11A2, GP1BA. |
| Melanogenesis | 23 | 0.033905 | ADCY2, LEF1, GNAQ, POMC, FZD9, WNT2B, PLCB2, WNT16, CREB3L2, PRKACG, WNT7A, EDN1, WNT6, WNT8B, ADCY4, WNT5B, TYR, MITF, WNT1, WNT7B, GNAO1, FZD10, WNT4. |
| Basal cell carcinoma | 15 | 0.036176 | AXIN1, LEF1, WNT2B, FZD9, WNT16, WNT7A, WNT6, WNT8B, SHH, APC, WNT5B, WNT1, WNT7B, FZD10, WNT4. |
| ABC transporters - General | 12 | 0.068373883 | ABCA13, ABCC11, ABCA6, ABCB8, ABCA9, ABCD4, ABCB1, ABCB5, TAP2, ABCA5, ABCC9, ABCG2, |
| Axon guidance | 27 | 0.09116048 | PAK3, ABLIM1, EPHA8, SEMA4G, LIMK2, SEMA3E, NTN4, EPHB6, ROBO1, ABLIM2, PAK6, EFNA3, EFNA2, ABLIM3, ARHGEF12, EPHA3, EPHA5, NFATC1, SEMA6C, PTK2, EPHA4, NFATC3, ITGB1, EFNB1, SLIT2, CXCL12, PLXNB3, |
| Calcium signaling pathway | 34 | 0.095851871 | SLC8A2, GNA15, ADCY2, RYR3, GNAQ, P2RX3, PLCB2, CACNA1H, PLCG2, PRKACG, EDNRA, ADCY4, PLCD1, ERBB2, GRIN1, ADRB1, CACNA1A, ERBB3, PLCE1, LHCGR, CACNA1S, CACNA1E, P2RX7, ATP2B2, CACNA1C, GRPR, PLN, CAMK4, CACNA1I, PTGER3, GNA14, PTGER1, TACR1, CHRM1, |

## Table 4B. Enriched KEGG pathways of upregulated genes with miR-181b inhibition in HeLa cells.

| **Term** | **Count** | **PValue** | **Genes** |
| --- | --- | --- | --- |
| MAPK signalling pathway | 54 | 0.005573 | STMN1, FGFR1, IL1B, FGF3, FGF5, FGF12, PRKACG, CACNA2D1, MAPK11, FGF20, CACNB2, EGFR, PRKCG, CACNB1, PPM1A, AKT2, MAP2K2, CASP3, MAP3K4, GNG12, MAPK7, MAP4K3, RASGRP3, FOS, MAP2K3, MAP3K14, DUSP10, PLA2G10, MAX, MAP2K6, RASA2, RPS6KA3, NF1, RASGRP1, SOS1, MAPK14, GNA12, TGFB1, NR4A1, IL1A, TP53, MAPKAPK2, DUSP4, FLNA, ACVR1B, CRK, CACNA1C, BDNF, DUSP7, PRKACB, TGFB2, PPM1B, NTRK2, MRAS. |
| Focal adhesion | 42 | 0.012476 | ILK, CTNNB1, PDPK1, MYLPF, SRC, COL6A3, ITGA4, PAK6, PIK3R5, GRLF1, ERBB2, SOS1, FYN, PPP1CA, PIK3CB, LAMB4, EGFR, PRKCG, COL4A6, SHC1, THBS1, ROCK1, VEGFC, ITGB4, AKT2, LAMA2, BAD, COL5A3, TNN, ITGA9, PARVB, BIRC2, TNXB, FLNA, ITGB1, ACTB, CRK, ITGAV, PAK7, VASP, MYLK, ROCK2. |
| Non-small cell lung cancer | 15 | 0.0149 | AKT2, PDPK1, RASSF1, BAD, MAP2K2, CDKN2A, TGFA, TP53, PIK3R5, RB1, ERBB2, SOS1, PIK3CB, EGFR, PRKCG. |
| Pancreatic cancer | 19 | 0.015873 | AKT2, TGFB1, BAD, CDKN2A, TGFA, TP53, RELA, ACVR1B, PIK3R5, RB1, STAT3, JAK1, ERBB2, PIK3CB, EGFR, ARHGEF6, TGFB2, RAD51, VEGFC. |
| Apoptosis | 21 | 0.016688 | PRKAR1B, AKT2, BAD, IL1A, IL1B, CASP3, CASP7, TP53, BIRC2, RELA, PRKAR2A, PRKACG, CASP6, PIK3R5, CASP8, MAP3K14, PIK3CB, CASP10, BAX, PRKAR1A, PRKACB. |
| Endometrial cancer | 14 | 0.024717 | ILK, AKT2, AXIN1, PDPK1, CTNNB1, BAD, MAP2K2, TP53, PIK3R5, ERBB2, SOS1, PIK3CB, EGFR, TCF7. |
| Bladder cancer | 12 | 0.025154 | RB1, RASSF1, ERBB2, MDM2, MAP2K2, ECGF1, CDKN2A, TP53, DAPK1, EGFR, THBS1, VEGFC. |
| ECM-receptor interaction | 21 | 0.027076 | HSPG2, CD36, ITGB4, LAMA2, COL5A3, TNN, ITGA9, GP6, SDC3, TNXB, ITGB1, COL6A3, ITGA4, GP5, ITGAV, SV2A, LAMB4, COL4A6, AGRN, CD44, THBS1. |
| Prostate cancer | 20 | 0.044544 | AKT2, FGFR1, CTNNB1, PDPK1, BAD, MAP2K2, TGFA, TP53, RELA, INSRR, PIK3R5, RB1, ERBB2, MDM2, SOS1, PIK3CB, KLK3, EGFR, TCF7, EP300. |
| Dentatorubropallidoluysian atrophy (DRPLA) | 6 | 0.056493634 | CASP8, CASP3, ATN1, CASP7, BAIAP2, MAGI1, |
| Regulation of actin cytoskeleton | 41 | 0.056973092 | FGFR1, LIMK2, FGF3, FGF5, MYLPF, FGF12, ARPC2, ITGA4, PAK6, PIK3R5, GRLF1, FGF20, SOS1, PFN3, PPP1CA, BAIAP2, PIK3CB, EGFR, ARHGEF6, GNA12, ROCK1, PFN2, ITGB4, SSH1, ITGA9, MAP2K2, WASF1, GNG12, ITGB1, ACTB, CRK, ITGAV, PAK7, ARPC4, PIP5K3, WASF2, PFN1, ARHGEF1, MRAS, MYLK, ROCK2, |
| ErbB signaling pathway | 19 | 0.064356961 | ABL2, AKT2, BAD, MAP2K2, RPS6KB2, TGFA, SRC, PAK6, CRK, PIK3R5, ERBB4, ERBB2, PAK7, SOS1, PIK3CB, CAMK2D, EGFR, PRKCG, SHC1, |
| Adherens junction | 17 | 0.066816727 | FGFR1, CTNNB1, PVRL1, WASF1, SRC, PTPN6, ACVR1B, ACTB, SNAI2, ERBB2, CSNK2A1, FYN, BAIAP2, WASF2, EGFR, TCF7, EP300, |
| Chronic myeloid leukemia | 17 | 0.066816727 | AKT2, TGFB1, BAD, MAP2K2, CDKN2A, TP53, RELA, ACVR1B, CRK, PIK3R5, RB1, MDM2, SOS1, PIK3CB, SHC1, TGFB2, CTBP2, |
| Melanoma | 16 | 0.071509001 | AKT2, FGFR1, BAD, MAP2K2, FGF3, FGF5, CDKN2A, TP53, FGF12, PIK3R5, RB1, FGF20, MDM2, MITF, PIK3CB, EGFR, |
| GnRH signaling pathway | 20 | 0.086082491 | PLA2G10, GNAQ, MAP2K2, MAP3K4, MAP2K6, SRC, MAPK7, PRKACG, PLCB3, MAPK11, MMP14, MAP2K3, CACNA1C, SOS1, PTK2B, CAMK2D, EGFR, MAPK14, ITPR2, PRKACB, |
| Glioma | 14 | 0.09124275 | AKT2, MAP2K2, CDKN2A, TGFA, TP53, PIK3R5, RB1, MDM2, SOS1, PIK3CB, CAMK2D, EGFR, PRKCG, SHC1, |
| PPAR signaling pathway | 16 | 0.096453028 | CD36, ILK, PDPK1, UBB, APOA5, SLC27A6, APOA1, ACSL5, SCP2, ACOX1, CYP4A11, ACADM, CYP4A22, CYP8B1, CYP7A1, CPT1B, |
| Acute myeloid leukemia | 13 | 0.097911393 | AKT2, RUNX1T1, BAD, RPS6KB2, MAP2K2, JUP, RELA, PIK3R5, STAT3, SOS1, PIK3CB, RARA, TCF7, |

## Table 5A. Enriched KEGG pathways of downregulated genes with miR-181b over-expression in SH-SY5Y cells.

| **Term** | **Count** | **PValue** | **Genes** |
| --- | --- | --- | --- |
| Neuroactive ligand-receptor interaction | 41 | 2.31E-05 | CALCR, GH2, GABRB1, CHRM2, GLRA1, FPRL2, LEPR, P2RX3, UTS2R, GABRB3, GRIA2, FPRL1, HRH4, GRM6, GABRA6, GRM7, GRIK1, SSTR5, SSTR1, GRIA1, GPR23, NMUR1, GLRA2, NPBWR1, EDG2, CTSG, GRIK4, OPRL1, GALR2, SSTR3, MC2R, FPR1, GRIN2C, LHB, GRIN2A, C5AR1, HCRTR1, NPY1R, DRD5, GZMA, PTGER3. |
| Cytokine-cytokine receptor interaction | 40 | 6.34E-05 | GH2, TNFSF8, IL1B, IL28A, LEPR, CCR3, LTA, TNFSF13B, CCR5, MPL, CCL13, IL6R, TNFRSF14, IL1RAP, IL12RB1, IL22, PDGFB, IL2RG, TNFRSF9, CXCL16, LIF, TNFRSF4, CCL1, IL28RA, CCL14, CCL8, IL21R, IFNA21, IL19, CCL27, LTB, IL24, ACVR1B, FLT3, INHBA, CCL21, CXCL10, CCL25, TGFB2, TPO. |
| ABC transporters - General | 9 | 0.026228 | ABCC11, ABCA8, ABCA12, ABCG1, ABCA2, ABCC6, ABCG5, ABCB11, CFTR. |
| Complement and coagulation cascades | 11 | 0.049827 | MASP1, C5AR1, C2, F8, FGG, KNG1, FGA, F5, SERPIND1, C4BPA, SERPINF2. |
| Jak-STAT signaling pathway | 19 | 0.064611099 | GH2, IL21R, IL13RA2, IFNA21, IL28A, IL19, LEPR, PIK3R1, IL24, SOCS7, MPL, IL6R, JAK3, IL12RB1, IL22, IL2RG, LIF, TPO, IL28RA, |

## Table 5B. Enriched KEGG pathways of upregulated genes with miR-181b inhibition in SH-SY5Y cells.

| **Term** | **Count** | **PValue** | **Genes** |
| --- | --- | --- | --- |
| Neuroactive ligand-receptor interaction | 39 | 0.007347 | GABRB1, P2RY6, VIPR2, CHRM2, EDG5, CGA, HTR4, GRM6, GABRQ, GABRA6, GRIK1, TBXA2R, LTB4R2, OPRM1, GRIA1, HTR1F, GALR3, EDG2, P2RY13, GABRG3, GRM3, FSHR, SCTR, GRIN3A, PTGER2, C3AR1, P2RY10, F2, TSHR, MC4R, GABRA5, GRM5, FSHB, HTR7, GRIN2B, NR3C1, CRHR2, HCRTR1, TRPV1. |
| Cell Communication | 24 | 0.008417 | ITGB4, COL17A1, KRT1, KRT24, ITGA6, GJB1, TNR, COL6A3, CHAD, KRT23, KRT15, RELN, LAMB3, THBS2, GJA10, KRT6C, KRT13, DSG1, KRT6B, KRT6A, GJA4, KRT9, COL11A2, LAMA3. |
| Cytokine-cytokine receptor interaction | 38 | 0.013938 | CCL20, IL13, IL10RA, TNFSF13B, CCR5, CCL15, TNFRSF19, IFNW1, IL1R1, PF4, IL9R, CCL16, NGFR, PDGFB, IL15RA, EGFR, CXCL11, TNFRSF11A, TNFRSF11B, CXCL5, IL21R, IL19, FLT1, IL21, LTB, FLT4, IL8RB, IL1R2, HGF, EDA, CCL26, TGFBR2, INHBE, TNFRSF13B, CCL19, BMP7, CXCL12, IL2RA. |
| MAPK signalling pathway | 38 | 0.016604 | PPP5C, STMN1, FGF21, ACVR1C, FGF3, FGF8, MAP2K6, FGF5, PRKACG, NF1, MKNK1, IL1R1, FGF20, PDGFB, PTPRR, FGF2, EGFR, PRKCG, CACNB1, NTRK1, PRKY, MAPK10, MAPK7, AKT1, CACNA1E, CACNB4, RASGRF1, IL1R2, FGF1, CACNG5, FLNC, TGFBR2, MAP2K3, MAPT, BDNF, CACNA1I, PLA2G2E, PRKACA. |
| Melanoma | 14 | 0.018615 | FGF21, FGF3, FGF8, FGF5, CDKN2A, AKT1, FGF1, HGF, PIK3R5, FGF20, MDM2, PDGFB, FGF2, EGFR. |
| GnRH signalling pathway | 17 | 0.024264 | PRKY, MAPK10, MAP2K6, MAPK7, CGA, PRKACG, MMP2, CALML3, ADCY4, FSHB, MMP14, MAP2K3, PTK2B, EGFR, PLA2G2E, PRKACA, PRKCD. |
| Hedgehog signalling pathway | 12 | 0.024772 | DHH, PRKY, LRP2, CSNK1G1, WNT11, CSNK1G3, PRKACG, BMP7, WNT10A, PRKACA, BMP6, WNT9B. |
| Calcium signalling pathway | 26 | 0.041776 | CHRM2, MLCK, PLCG2, PRKACG, HTR4, ADCY4, TBXA2R, SPHK1, LTB4R2, PHKG1, EGFR, TNNC1, PRKCG, ERBB3, RYR2, PRKY, PLCD3, CACNA1E, GRM5, CALML3, HTR7, PTK2B, CACNA1I, CAMK4, GNA14, PRKACA. |
| Hematopoietic cell lineage | 15 | 0.043241 | CD34, CD5, DNTT, ITGA6, ITGA4, IL1R2, GYPE, IL1R1, IL9R, MME, CD7, ITGA2, CD44, CD3G, IL2RA. |
| Cell adhesion molecules (CAMs) | 20 | 0.053341738 | PTPRF, CD34, CD226, PVRL1, CLDN7, ICOSLG, NRXN3, SELL, CD6, CLDN2, ITGA6, ITGA4, CD80, ICAM1, CLDN18, CLDN11, CLDN20, HLA-DQB2, PTPRC, L1CAM, |
| Fructose and mannose metabolism | 9 | 0.055270361 | MTMR1, ALDOA, HK1, PFKFB1, RDH12, HK3, HSD3B7, KHK, PFKL, |
| ECM-receptor interaction | 15 | 0.055526507 | ITGB4, ITGB6, ITGA6, TNR, ITGA4, COL6A3, CHAD, RELN, LAMB3, THBS2, ITGA2, SV2B, CD44, COL11A2, LAMA3, |
| Focal adhesion | 28 | 0.064697489 | FIGF, MYLPF, MLCK, ITGA6, ITGA4, COL6A3, PARVG, PIK3R5, MYL9, LAMB3, RELN, PDGFB, EGFR, PRKCG, ITGA2, LAMA3, ITGB4, ITGB6, MAPK10, FLT1, AKT1, TNR, CHAD, HGF, THBS2, FLNC, ARHGAP5, COL11A2, |
| Leukocyte transendothelial migration | 18 | 0.076644653 | CLDN7, MYLPF, CLDN2, PLCG2, ITGA4, MMP2, MYL9, PIK3R5, ICAM1, CLDN18, CLDN11, ARHGAP5, RAPGEF3, CLDN20, RHOH, PTK2B, PRKCG, CXCL12, |
| ABC transporters - General | 9 | 0.077627796 | ABCC11, ABCG1, ABCG4, ABCA10, ABCC3, ABCA5, ABCD1, ABCC9, ABCC2, |
| Renin-angiotensin system | 5 | 0.08431724 | ACE2, ACE, MME, LNPEP, CPA3, |
| Taste transduction | 10 | 0.088446305 | PRKY, TAS2R50, ACCN1, TAS2R10, TRPM5, PRKACG, PRKACA, TAS2R39, TAS2R49, ADCY4, |
| C21-Steroid hormone metabolism | 4 | 0.091137264 | CYP21A2, CYP11B2, AKR1C4, CYP11B1, |

## Table 6A. Enriched KEGG pathways of genes differentially expressed with both miR-181b over-expression and inhibition in HEK-293 cells.

| **Term** | **Count** | **PValue** | **Genes** |
| --- | --- | --- | --- |
| Neuroactive ligand-receptor interaction | 18 | 0.004033 | CHRM2, ADORA3, P2RY6, LEPR, EDG1, GABRA5, HRH4, GPR35, LTB4R2, GRIA1, HTR1F, GALR3, DRD1, GH1, HTR2A, CYSLTR2, EDNRB, HRH2, |
| Fc epsilon RI signalling pathway | 7 | 0.035186 | PLA2G10, MAPK10, IL3, AKT3, PIK3R1, PLA2G2E, IL5, |
| Hematopoietic cell lineage | 7 | 0.061998 | MME, IL3, GP9, KITLG, IL2RA, IL5, GYPE, |
| Jak-STAT signalling pathway | 10 | 0.064461 | IL3, LEPR, SPRY3, STAM2, AKT3, PIK3R1, IL10RA, GH1, IL2RA, IL5, |
| Melanoma | 6 | 0.077845 | CDKN2A, FGF2, FGF22, AKT3, PIK3R1, EGFR, |
| GnRH signalling pathway | 7 | 0.091017 | PLA2G10, MAPK10, MMP14, PTK2B, EGFR, PLA2G2E, CACNA1D, |
| Non-small cell lung cancer | 5 | 0.092815 | CDKN2A, AKT3, PIK3R1, EGFR, RXRG, |

## Table 6B. Enriched KEGG pathways of genes differentially expressed with both miR-181b over-expression and inhibition in HeLa cells.

| **Term** | **Count** | **PValue** | **Genes** |
| --- | --- | --- | --- |
| MAPK signalling pathway | 17 | 0.005951 | NR4A1, MAP3K4, FGF5, MAPKAPK2, DUSP4, FGF12, PRKACG, ACVR1B, MAPK11, CACNA2D1, MAP2K3, CACNA1C, BDNF, MAPK14, TGFB2, CACNB1, GNA12, |
| Taste transduction | 6 | 0.025157 | SCNN1B, TAS2R4, ACCN1, PRKACG, TAS2R45, TAS2R3, |
| GnRH signalling pathway | 7 | 0.070902 | MAPK11, GNAQ, MAP2K3, CACNA1C, MAP3K4, PRKACG, MAPK14, |
| Hedgehog signalling pathway | 5 | 0.099812 | CSNK1G1, WNT2B, WNT16, PRKACG, SHH, |

## Table 6C. Enriched KEGG pathways of genes differentially expressed with both miR-181b over-expression and inhibition in SH-SY5Y cells.

| **Term** | **Count** | **PValue** | **Genes** |
| --- | --- | --- | --- |
| Thyroid cancer | 3 | 0.07868 | PAX8, TCF7, NTRK1, |

## Table 6D. Enriched KEGG pathways of genes modulated by either miR-181b over-expression or inhibition across all three cell lines.

| **Term** | **Count** | **PValue** | **Genes** |
| --- | --- | --- | --- |
| Haematopoietic cell lineage | 11 | 0.001991 | CD34, CD5, DNTT, MME, IL1B, ITGA6, ITGA4, IL1R2, CD44, CD3G, IL2RA, |
| Cytokine-cytokine receptor interaction | 21 | 0.002985 | IL21R, TNFSF8, IL1B, LEPR, ACVR1B, IL1R2, CCR5, IFNW1, CCL13, PF4, TNFRSF14, IL12RB1, CCL21, TNFRSF9, CXCL10, EGFR, CCL25, CXCL12, TNFRSF4, CXCL5, IL2RA, |
| Melanoma | 9 | 0.006081 | FGF20, MDM2, FGF3, CDKN2A, FGF5, FGF12, FGF22, EGFR, PIK3R5, |
| MAPK signalling pathway | 20 | 0.0075 | IL1B, FGF3, FGF5, MAP2K6, FGF12, PRKACG, CACNA1E, ACVR1B, IL1R2, CACNG5, FGF20, MAP2K3, RASGRP1, MAPT, BDNF, FGF22, EGFR, CACNB1, GNA12, NTRK1, |
| Cell adhesion molecules (CAMs) | 11 | 0.032659 | CD34, HLA-F, CD6, HLA-DQB2, PTPRC, ITGA6, ITGA4, NFASC, NRXN2, L1CAM, ITGB2, |
| Regulation of actin cytoskeleton | 15 | 0.048463 | FGF3, FGF5, FGF12, ITGA6, ITGA4, ITGB2, PIK3R5, FGF20, BAIAP2, PIP5K3, FGF22, PPP1R12B, EGFR, GNA12, MYLK, |
| Acute myeloid leukemia | 6 | 0.069217387 | RUNX1T1, PML, RARA, TCF7, SPI1, PIK3R5, |

## Table 7. Enriched KEGG pathways of predicted miR-107 target genes.

| **Term** | **Count** | **PValue** | **Genes** |
| --- | --- | --- | --- |
| Wnt signaling pathway | 30 | 8.68E-05 | PPP2R5A, BTRC, PPP2R5D, WNT3A, CAMK2G, PPP2R5C, PPP3R1, CSNK2A2, MAP3K7, NFAT5, PPP3CB, NFATC4, PPP3CA, AXIN2, NFATC3, APC, PPP2R1A, TBL1XR1, CTBP2, VANGL2, CREBBP, FZD7, FZD6, DVL1, DVL1L1, FZD10, CCND2, LRP6, MAPK9, SIAH1, WNT7A |
| Fc gamma R-mediated phagocytosis | 21 | 3.10E-04 | MAP2K1, MARCKSL1, NCF1, WASF2, RPS6KB2, RAF1, RPS6KB1, PRKCE, WAS, CDC42, ARPC1B, PLA2G4A, CRKL, FCGR2B, ARPC2, ARPC5L, CFL1, WASL, PIK3R1, AKT3, PLA2G4D |
| Pathways in cancer | 50 | 3.40E-04 | FGF18, E2F3, WNT3A, FGF10, GLI3, CCNE1, CUL2, CDC42, CASP3, RARB, FGF2, AKT3, RET, BCR, CTBP2, RUNX1T1, CDK6, DVL1L1, CCDC6, CRKL, PDGFRA, PDGFRB, MAPK9, PIAS2, LAMC1, PIAS1, FGFR2, EGLN2, SUFU, ARNT, LAMB4, BCL2, RUNX1, AXIN2, FIGF, PIK3R1, APC, MAP2K1, CREBBP, ITGA2, RAF1, FZD7, DVL1, FZD6, NRAS, FZD10, RASSF5, ARAF, IKBKG, TCEB1, WNT7A |
| Regulation of actin cytoskeleton | 36 | 5.13E-04 | FGFR2, FGF18, ENAH, MRAS, WASF2, FGF10, VCL, ACTG1, CDC42, ITGAX, TIAM2, ARPC2, PAK3, FGF2, PIK3R1, APC, MAP2K1, BAIAP2, ITGA2, RAF1, MYH9, WAS, ITGA9, NRAS, ARPC1B, PPP1CA, CRKL, ARPC5L, CHRM1, ARAF, CFL1, PDGFRA, PDGFRB, CYFIP1, WASL, SLC9A1 |
| MAPK signaling pathway | 42 | 6.05E-04 | FGFR2, FGF18, TNF, MAPKAPK5, MRAS, CACNB1, PPP3R1, PPM1A, FGF10, CACNB2, MAP3K7, CDC42, CASP3, BDNF, MAP3K4, MAP3K3, PPP3CB, NFATC4, PPP3CA, FGF2, AKT3, MAP2K5, CACNA2D1, MAP2K1, MAP2K3, NF1, TAOK3, RAF1, FLNA, RPS6KA5, NRAS, PLA2G4A, RPS6KA3, CRKL, RASGRF1, GADD45G, IKBKG, PDGFRA, MAPK9, PDGFRB, MAPK7, CACNA1C |
| Axon guidance | 24 | 0.001359711 | GNAI3, PLXNA2, EFNB1, EFNB2, PPP3R1, EPHA1, EPHA3, CDC42, SEMA5B, NRAS, EPHA4, EPHA7, SEMA6D, UNC5A, PAK3, CFL1, NFAT5, PPP3CB, SEMA3D, NFATC4, SEMA4D, PPP3CA, NFATC3, SRGAP1 |
| Renal cell carcinoma | 16 | 0.001423396 | MAP2K1, CREBBP, EGLN2, RAF1, ARNT, NRAS, CUL2, CDC42, CRKL, PAK3, ARAF, TCEB1, RAPGEF1, FIGF, PIK3R1, AKT3 |
| Neurotrophin signaling pathway | 23 | 0.001834423 | MAP2K1, CAMK2G, YWHAB, RAF1, RPS6KA5, CDC42, NRAS, RPS6KA3, YWHAG, BDNF, CRKL, YWHAH, PRDM4, MAP3K3, BCL2, YWHAQ, MAPK9, MAPK7, RAPGEF1, PIK3R1, ARHGDIA, AKT3, MAP2K5 |
| D-Glutamine and D-glutamate metabolism | 4 | 0.002887466 | GLS2, GLUD2, GLUD1, GLS |
| Long-term potentiation | 15 | 0.003020405 | MAP2K1, CAMK2G, CREBBP, GRIN1, PPP3R1, RAF1, ITPR1, NRAS, PPP1CA, RPS6KA3, GRIA2, ARAF, PPP3CB, PPP3CA, CACNA1C |
| Focal adhesion | 31 | 0.004955702 | CAV3, COL2A1, VCL, CHAD, ACTG1, CDC42, LAMB4, PAK3, BCL2, ZYX, FIGF, RAPGEF1, AKT3, PIK3R1, MAP2K1, ITGA2, RAF1, FLNA, KDR, ITGA9, VWF, PPP1CA, CRKL, CCND2, RASGRF1, PDGFRA, PDGFRB, MAPK9, RELN, COL1A1, LAMC1 |
| Oocyte meiosis | 20 | 0.004985549 | PPP2R1A, MAP2K1, PPP2R5A, PPP2R5D, CAMK2G, BTRC, PPP2R5C, YWHAB, PPP3R1, AURKA, ITPR1, CCNE1, PPP1CA, RPS6KA3, YWHAG, YWHAH, YWHAQ, PPP3CB, PPP3CA, MAD2L2 |
| Prostate cancer | 17 | 0.006493164 | FGFR2, E2F3, CREB3, MAP2K1, CREBBP, RAF1, CREB5, NRAS, CCNE1, BCL2, ARAF, IKBKG, PDGFRA, PDGFRB, CREB3L3, PIK3R1, AKT3 |
| VEGF signaling pathway | 15 | 0.007565353 | MAP2K1, PPP3R1, RAF1, KDR, CDC42, NRAS, SH2D2A, PLA2G4A, NFAT5, PPP3CB, NFATC4, PPP3CA, NFATC3, PIK3R1, AKT3 |
| B cell receptor signaling pathway | 15 | 0.007565353 | MAP2K1, IFITM1, PPP3R1, RAF1, NRAS, CD19, FCGR2B, IKBKG, NFAT5, PPP3CB, NFATC4, PPP3CA, NFATC3, PIK3R1, AKT3 |
| Colorectal cancer | 16 | 0.00880689 | MAP2K1, RAF1, FZD7, FZD6, DVL1, DVL1L1, FZD10, CASP3, BCL2, ARAF, PDGFRA, PDGFRB, MAPK9, AXIN2, PIK3R1, AKT3, APC |
| T cell receptor signaling pathway | 19 | 0.009006138 | TNF, MAP2K1, CD8A, PPP3R1, RAF1, MAP3K7, CDC42, NRAS, PAK3, IKBKG, NFAT5, PPP3CB, MAPK9, NFATC4, PPP3CA, NFATC3, PIK3R1, AKT3, CD28 |
| Acute myeloid leukemia | 12 | 0.015139614 | NRAS, MAP2K1, ARAF, IKBKG, RUNX1T1, PIM1, RAF1, RPS6KB2, RPS6KB1, RUNX1, AKT3, PIK3R1 |
| Amyotrophic lateral sclerosis (ALS) | 11 | 0.020799975 | PRPH, GPX1, CASP3, TNF, GRIA2, BCL2, MAP2K3, GRIN1, PPP3CB, PPP3R1, PPP3CA |
| Insulin signaling pathway | 21 | 0.021351407 | PRKAG3, MAP2K1, FLOT2, PHKG2, PRKAG2, FBP1, RPS6KB2, RAF1, PDE3B, RPS6KB1, PPARGC1A, NRAS, PPP1CA, CRKL, ARAF, FASN, MAPK9, TRIP10, RAPGEF1, PIK3R1, AKT3 |
| Ubiquitin mediated proteolysis | 21 | 0.024678533 | UBE2A, BTRC, UBE4B, UBE2J1, HERC2, UBE2Q1, UBE2R2, RFWD2, UBE2D4, CUL2, UBE2E3, UBE2D3, CUL5, FBXW7, UBE2D2, AIRE, RHOBTB2, PIAS2, SIAH1, PIAS1, TCEB1 |
| Melanoma | 13 | 0.027120535 | FGF18, E2F3, MAP2K1, RAF1, FGF10, CDK6, NRAS, ARAF, PDGFRA, PDGFRB, FGF2, PIK3R1, AKT3 |
| Cell cycle | 19 | 0.036117917 | E2F3, CREBBP, YWHAB, CDK6, CHEK1, CDK7, WEE1, CDC25A, MCM5, ORC1L, CCNE1, YWHAG, RAD21, MCM7, YWHAH, CCND2, GADD45G, YWHAQ, MAD2L2 |
| Chronic myeloid leukemia | 13 | 0.039663628 | E2F3, BCR, CTBP2, MAP2K1, RAF1, CDK6, NRAS, CRKL, IKBKG, ARAF, RUNX1, PIK3R1, AKT3 |
| Adherens junction | 13 | 0.047262051 | BAIAP2, CREBBP, WASF2, CTNND1, WAS, VCL, MAP3K7, ACTG1, CSNK2A2, CDC42, PVRL4, TJP1, WASL |
| Long-term depression | 12 | 0.048748169 | NRAS, PPP2R1A, PLA2G4A, GNAO1, GNAI3, MAP2K1, GRIA2, C7ORF16, ARAF, RAF1, PRKG1, ITPR1 |
| Calcium signaling pathway | 24 | 0.052308016 | TRPC1, DRD1, SLC8A2, BST1, DRD5, PHKG2, CAMK2G, GRIN1, PPP3R1, HTR4, VDAC2, ITPR1, ATP2B1, ATP2B2, PLCE1, ADRB2, CHRM1, LTB4R2, PDGFRA, PPP3CB, PDGFRB, PPP3CA, CACNA1C, HTR2A |
| Non-small cell lung cancer | 10 | 0.05587011 | NRAS, E2F3, RASSF5, MAP2K1, ARAF, RAF1, CDK6, RARB, AKT3, PIK3R1 |
| Glioma | 11 | 0.060063024 | NRAS, E2F3, MAP2K1, CAMK2G, ARAF, PDGFRA, PDGFRB, RAF1, CDK6, AKT3, PIK3R1 |
| Basal cell carcinoma | 10 | 0.061624347 | DVL1L1, FZD10, WNT3A, AXIN2, WNT7A, GLI3, SUFU, FZD7, FZD6, DVL1, APC |
| Tight junction | 19 | 0.064281714 | CLDN8, PPP2R1A, GNAI3, HCLS1, MRAS, CRB3, CASK, CLDN11, MYH9, PRKCE, LLGL2, CSNK2A2, ACTG1, CDC42, NRAS, TJP1, AKT3, MYH7B, SPTAN1 |
| Melanogenesis | 15 | 0.067817982 | GNAI3, GNAO1, MAP2K1, CREB3, WNT3A, CAMK2G, CREBBP, RAF1, FZD7, FZD6, DVL1, DVL1L1, NRAS, FZD10, CREB3L3, WNT7A |
| Lysosome | 17 | 0.068547939 | LAPTM4A, AP3S1, CD164, M6PR, MANBA, GNS, CTSK, GNPTAB, IDS, TPP1, AP3M1, IGF2R, SMPD1, ATP6V0D1, GGA3, CTSG, IDUA |
| Pathogenic Escherichia coli infection | 10 | 0.074231061 | ACTG1, CDC42, ARPC1B, ARPC2, HCLS1, ARPC5L, YWHAQ, TLR4, WASL, WAS |
| RNA degradation | 10 | 0.074231061 | EXOSC10, EXOSC8, EXOSC9, EXOSC6, WDR61, CNOT6L, RQCD1, CNOT1, EXOSC1, XRN2 |
| Small cell lung cancer | 13 | 0.081523454 | E2F3, ITGA2, CDK6, LAMB4, CCNE1, BCL2, IKBKG, PIAS2, LAMC1, RARB, PIAS1, PIK3R1, AKT3 |
| Adipocytokine signaling pathway | 11 | 0.084235006 | PRKAG3, CPT1B, TNF, ACSL1, PRKAG2, IKBKG, MAPK9, ACSL4, PPARGC1A, AKT3, CAMKK1 |
| Hypertrophic cardiomyopathy (HCM) | 13 | 0.087435608 | PRKAG3, CACNA2D1, TNF, MYL3, PRKAG2, CACNB1, ITGA2, CACNB2, TPM1, ACTG1, ITGA9, SGCD, CACNA1C |
| p53 signaling pathway | 11 | 0.091086765 | RFWD2, CCNE1, PPM1D, CASP3, CCND2, BAI1, GADD45G, SIAH1, CDK6, CHEK1, SESN1 |

## Table 8A. Significantly enriched KEGG pathways of downregulated genes with miR-107 over-expression in HEK-293 cells.

| **Term** | **Count** | **PValue** | **Genes** |
| --- | --- | --- | --- |
| Neuroactive ligand-receptor interaction | 65 | 3.09E-08 | F2RL2, CSH1, GPR83, OPRM1, ADORA3, GABRB3, GRIK1, LEPR, GABRB1, LHCGR, GRIK4, GNRHR, GHRHR, AGTR1, EDNRB, HTR1B, GRIN2B, LTB4R, GALR3, GALR2, CALCRL, GLP2R, HTR1E, GABRG1, PTGER1, GABRG2, PTGER2, SSTR5, GRM3, GRM2, CHRM4, CHRM2, GRM7, HTR7, GRM6, MC2R, CTSG, DRD1, DRD2, PPYR1, FPR1, PRSS1, BDKRB1, BDKRB2, HCRTR2, ADRB3, HRH2, P2RY2, CNR1, PRSS3, MAS1, GABRQ, GABRA2, GABRA5, GRIN1, LEP, GH1, P2RY10, P2RX7, P2RX1, GRIA2, TBXA2R, AVPR1A, MTNR1A, HTR2A |
| Systemic lupus erythematosus | 32 | 8.44E-07 | C7, LOC340096, HIST1H2AA, HIST1H4K, HIST1H2AG, C3, HLA-DRB3, IL10, HIST1H2BM, GRIN2B, HIST1H2BI, HIST3H3, HIST3H2BB, HLA-DOA, HIST1H4I, HIST1H4G, CD28, HIST1H2BA, HIST1H2BC, HIST1H2BD, C4A, HIST1H2BF, ACTN2, H2AFJ, HLA-DQA2, HIST2H3C, HLA-DQA1, C8A, C1QA, C8B, CD86, CD80, FCGR2B, FCGR2C, HIST1H3C, FCGR2A, HIST1H2AM, HIST1H3G, HIST1H3H, CTSG |
| Cytokine-cytokine receptor interaction | 59 | 1.01E-05 | IL9R, ACVRL1, PDGFB, IL6ST, LEPR, IL19, CXCR3, IL15, IL10, CXCL10, ACVR1B, TNFRSF11A, IL1RAP, IL15RA, CSF2RB, XCR1, CSF2RA, IL26, OSM, IFNAR2, TNFRSF10C, CCR7, IL20RA, CCR3, PDGFRA, IL12B, NGFR, CSF3, CCL1, CCL3, IL1R1, CXCL5, CTF1, CSF1, KITLG, TNFRSF8, PF4, CNTFR, CCL28, CCL27, CCL24, CCL25, IL12RB1, CCL23, CCL21, IL10RA, EGF, FLT1, FLT4, CCL19, CCL17, LEP, GH1, CNTF, CCL14, TNFSF11, BMPR1B, MPL, XCL2 |
| Cell adhesion molecules (CAMs) | 32 | 4.19E-04 | CLDN6, HLA-DRB3, CDH1, ITGB2, CLDN10, PVRL1, ITGB8, ICOS, CD22, ESAM, CD4, HLA-DOA, NEGR1, SPN, CD28, NRXN3, NFASC, CTLA4, NLGN3, ITGA4, NRXN1, HLA-DQA2, PDCD1LG2, HLA-G, HLA-DQA1, NCAM1, CD86, CD80, PECAM1, CLDN1, CNTN1, SELE |
| Intestinal immune network for IgA production | 16 | 7.77E-04 | HLA-DRB3, ITGA4, IL15, PIGR, CCL28, HLA-DQA2, CCL27, HLA-DQA1, IL10, CCL25, CD86, CD80, ICOS, IL15RA, HLA-DOA, CD28 |
| Viral myocarditis | 20 | 0.001049119 | PRF1, HLA-DRB3, MYH4, ITGB2, MYH6, HLA-DQA2, HLA-G, HLA-DQA1, LAMA2, CD86, CD80, RAC2, DMD, CASP8, SGCD, MYH13, ABL1, HLA-DOA, ABL2, CD28 |
| Focal adhesion | 42 | 0.001221372 | CAV3, PDGFB, ERBB2, ITGB4, ITGA10, COL2A1, VTN, MYL9, LAMB4, LAMB3, PTK2, RAC2, ITGB8, COMP, COL6A3, COL11A2, PIK3R3, EGF, RAPGEF1, THBS2, AKT3, FN1, PARVG, PRKCA, FLT1, FLT4, IGF1, MYLK2, ACTN2, MAPK10, ITGA4, COL4A6, LAMA2, RASGRF1, ITGA7, COL1A2, PDGFRA, RELN, LAMC2, COL1A1, MYLK, PARVA |
| ECM-receptor interaction | 22 | 0.001513107 | ITGB4, ITGA10, VTN, COL2A1, ITGA4, COL4A6, LAMA2, LAMB4, LAMB3, GP6, ITGB8, COMP, ITGA7, COL6A3, COL1A2, LAMC2, GP1BA, RELN, COL1A1, COL11A2, THBS2, FN1 |
| Type I diabetes mellitus | 13 | 0.00483157 | PRF1, HLA-DRB3, PTPRN, HLA-DQA2, HLA-G, HLA-DQA1, CD86, CD80, INS, HSPD1, IL12B, HLA-DOA, CD28 |
| Allograft rejection | 11 | 0.012044312 | PRF1, CD86, CD80, HLA-DRB3, IL12B, HLA-DOA, HLA-DQA2, HLA-G, HLA-DQA1, IL10, CD28 |
| Renin-angiotensin system | 7 | 0.014963109 | LNPEP, AGTR1, MAS1, MME, CPA3, ENPEP, CTSG |
| Retinol metabolism | 14 | 0.015769155 | CYP3A4, CYP1A1, CYP2C9, CYP2C8, ADH1C, ADH6, ADH1A, RPE65, CYP2A13, LRAT, CYP26C1, UGT1A3, CYP26B1, UGT2B10, UGT2B28 |
| Metabolism of xenobiotics by cytochrome P450 | 15 | 0.01639124 | CYP3A4, GSTA3, CYP2F1, CYP1A1, CYP2C9, CYP2C8, ADH1C, ADH6, ALDH3B2, ADH1A, GSTT2, ALDH3A1, GSTM1, UGT1A3, UGT2B10, UGT2B28 |
| Chemokine signaling pathway | 35 | 0.018666099 | CCL1, CCL3, CXCL5, ADCY7, ADCY5, PF4, CXCR3, CCL28, CCL27, CXCL10, CCL24, CCL25, PTK2, CCL23, RAC2, CCL21, TIAM1, GNG3, PIK3R3, XCR1, PLCB1, AKT3, GNG7, NCF1, HCK, CCL19, ELMO1, CCL17, CCR7, CCL14, ARRB1, CCR3, GRK4, GNB3, XCL2 |
| Jak-STAT signaling pathway | 30 | 0.019881713 | CSH1, CSF3, IL9R, IL6ST, LEPR, CTF1, IL19, CNTFR, IL15, IL10, SPRY4, SPRY3, IL12RB1, IL10RA, IL15RA, CSF2RB, PIK3R3, AKT3, CSF2RA, SOCS3, IL26, LEP, OSM, IFNAR2, CBLC, GH1, CNTF, IL20RA, IL12B, MPL |
| Graft-versus-host disease | 11 | 0.021196791 | PRF1, CD86, CD80, HLA-DRB3, HLA-DOA, KLRD1, KIR2DL2, HLA-DQA2, HLA-G, HLA-DQA1, CD28 |
| Drug metabolism | 15 | 0.021620384 | CYP3A4, GSTA3, CYP2C9, CYP2C8, ADH1C, ADH6, ALDH3B2, GSTT2, ADH1A, ALDH3A1, GSTM1, CYP2A13, UGT1A3, AOX1, UGT2B10, UGT2B28 |
| Calcium signaling pathway | 33 | 0.022066541 | GNA15, DRD1, ADCY7, ERBB2, LHCGR, BDKRB1, BDKRB2, ATP2B2, ADRB3, AGTR1, EDNRB, HRH2, PDE1C, CALML5, PLCB1, PRKCA, PTGER1, BST1, GRIN1, CACNA1I, MYLK2, P2RX7, P2RX1, CHRM2, HTR7, RYR3, PDGFRA, AVPR1A, TBXA2R, CACNA1E, CACNA1C, MYLK, HTR2A |
| Complement and coagulation cascades | 16 | 0.024717262 | MBL2, C7, CR1, MASP1, C4A, MASP2, C3, F13A1, BDKRB1, BDKRB2, C1QA, C8A, F13B, C8B, FGG, FGB |
| Maturity onset diabetes of the young | 8 | 0.032243852 | GCK, ONECUT1, INS, PAX4, MAFA, NEUROG3, NR5A2, NKX6-1 |
| Drug metabolism | 11 | 0.040128827 | CYP3A4, CYP2A13, UMPS, CES1, UGT1A3, UPB1, UPP1, UPP2, DPYS, UGT2B10, UGT2B28 |
| Melanogenesis | 20 | 0.042092693 | PRKCA, WNT10A, WNT16, TYRP1, GNAO1, ADCY7, WNT3A, ADCY5, EDN1, MITF, KITLG, FZD5, EDNRB, WNT1, FZD10, TYR, CALML5, PLCB1, WNT6, WNT8B |
| Autoimmune thyroid disease | 12 | 0.052818316 | TG, PRF1, CD86, CD80, HLA-DRB3, CTLA4, HLA-DOA, HLA-DQA2, HLA-G, HLA-DQA1, IL10, CD28 |
| Linoleic acid metabolism | 8 | 0.056793627 | CYP3A4, ALOX15, CYP2C9, AKR1B10, CYP2C8, PLA2G12B, PLA2G2E, PLA2G2F |
| Axon guidance | 24 | 0.057192551 | ABLIM1, ABLIM2, LIMK1, PLXNA2, EFNA2, EFNA3, NTNG1, NTN1, EPHA3, SEMA5B, PTK2, RAC2, SEMA4G, SEMA6D, RGS3, ROBO1, SEMA3F, SEMA3D, SEMA4B, SEMA3B, UNC5D, NFATC4, EFNA5, ABL1 |
| Fructose and mannose metabolism | 9 | 0.059309503 | KHK, GMPPB, MTMR1, PFKFB3, AKR1B10, GMPPA, HK1, TNNI3K, FBP2 |
| Hypertrophic cardiomyopathy (HCM) | 17 | 0.068173713 | PRKAG3, PRKAB2, CACNG5, CACNB1, ITGB4, ITGA10, IGF1, CACNB2, MYH6, ITGA4, TNNI3, LAMA2, ITGB8, DMD, ITGA7, SGCD, CACNA1C |
| Hematopoietic cell lineage | 17 | 0.074347681 | CSF3, IL1R1, CR1, IL9R, CD3D, CSF1, HLA-DRB3, KITLG, MME, CD1B, CD1A, ITGA4, DNTT, CD22, GP1BA, CD4, CSF2RA |
| Keratan sulfate biosynthesis | 5 | 0.092130749 | B4GALT2, CHST6, B3GNT7, CHST2, CHST4 |

## Table 8B. Significantly enriched KEGG pathways of upregulated genes with miR-107 inhibition in HEK-293 cells.

| **Term** | **Count** | **PValue** | **Genes** |
| --- | --- | --- | --- |
| Prostate cancer | 32 | 0.001803135 | HSP90AB1, FGFR1, PDGFB, GRB2, INSRR, CTNNB1, CCNE2, PDPK1, INS, SOS1, CREB3L1, TGFA, PIK3CA, CREB3L4, CREB3L3, PDGFD, EGFR, PIK3CG, TCF7, AR, HSP90AA1, BRAF, PIK3CB, MAP2K2, CREB1, RELA, CREBBP, TP53, RB1, BAD, EP300, PDGFRA |
| Hematopoietic cell lineage | 29 | 0.008510306 | IL1R1, CD8A, CSF1, KITLG, ANPEP, KIT, GP9, CD44, DNTT, HLA-DRB4, CD2, TPO, IL1B, CD4, CSF1R, IL3, CD3G, CR2, CD3E, ITGA2, ITGA4, IL6R, IL11RA, CD38, CD37, CD19, CD34, CD33, CD14 |
| Neurotrophin signaling pathway | 38 | 0.013059246 | ZNF274, GRB2, IRAK4, IRAK3, BDNF, MAP3K3, SOS1, GAB1, CAMK2D, PIK3CA, SHC1, CALML5, SHC4, PIK3CG, IRS2, BRAF, MAP2K2, PIK3CB, RELA, YWHAB, TP53, MAPK11, BAD, MAPK10, KIDINS220, IRS1, YWHAE, TP73, NTRK3, YWHAG, CAMK4, PSEN1, MAPK14, BAX, NTRK1, RAP1A, MAPK9, CRK |
| Pathways in cancer | 86 | 0.018211476 | HSP90AB1, FGF5, FGF18, PDGFB, MMP9, MITF, PPARG, FGF13, GLI2, CTNNB1, TGFB2, CCNE2, FOS, MAX, CDKN2A, CDKN2B, CASP8, PAX8, PIK3CA, TGFA, HHIP, FGF2, PIK3CG, EGFR, AR, HSP90AA1, CTBP2, BRAF, PIK3CB, RELA, TP53, RUNX1T1, RB1, FGF21, FGF20, DAPK1, RAD51, CTNNA2, JUP, VEGFC, EP300, HIF1A, PDGFRA, MAPK9, WNT5A, FGFR1, CKS1B, APC2, GRB2, PML, TFG, KITLG, EGLN1, KIT, ITGB1, SOS1, ITGAV, TRAF4, AXIN1, FN1, CSF1R, CEBPA, DVL3, TCF7, COL4A2, MSH2, MAP2K2, CREBBP, BRCA2, ITGA2, BAD, MAPK10, BIRC2, COL4A6, STAT3, FZD7, DVL1, LAMA1, RASSF5, LAMA4, BAX, RASSF1, NTRK1, JAK1, TCEB1, CRK |
| Cell cycle | 37 | 0.024630531 | MAD1L1, E2F5, CDC14A, CDC14B, DBF4, PKMYT1, CHEK2, ANAPC11, SFN, PTTG1, TGFB2, CCNE2, MCM7, CDKN2A, CDKN2B, CDKN2C, BUB1, STAG2, RBL2, CCNH, CREBBP, YWHAB, TP53, RB1, MCM2, MCM3, MCM4, YWHAE, WEE1, ATM, YWHAG, MAD2L1, EP300, CCND2, PCNA, ANAPC7, GADD45A |
| Fructose and mannose metabolism | 13 | 0.03988031 | ALDOA, SORD, PFKL, PFKFB3, ALDOB, HK2, HK1, FBP2, TPI1, MPI, AKR1B1, GMPPA, FPGT |
| DNA replication | 13 | 0.060423518 | LIG1, MCM2, MCM3, MCM4, RPA4, POLD3, PRIM1, RPA1, MCM7, RFC1, RFC2, PCNA, FEN1 |
| MAPK signaling pathway | 68 | 0.061797739 | FGF18, FGF5, ZAK, PDGFB, FGF13, DAXX, PRKX, TGFB2, FOS, MAX, BDNF, MAP3K4, MAPT, IL1B, FGF2, MAP2K6, EGFR, BRAF, RELA, CACNG7, TP53, CACNG5, CACNG4, CACNG3, FGF21, CACNG1, FGF20, FLNA, MAP4K3, MAP4K4, PDGFRA, MAPK9, PLA2G2E, GADD45A, FGFR1, IL1R1, GRB2, MRAS, MKNK2, PPM1A, CACNB1, DUSP10, MKNK1, PPM1B, CACNB4, GNG12, MAP3K3, HSPA2, RASGRP3, SOS1, RASA2, PLA2G10, MAP2K2, MAP2K4, MAPK11, MAPK10, DUSP4, PLA2G4A, MAPK14, NTRK1, MAPK8IP2, CACNA1G, CACNA1H, RAP1A, CACNA1E, CRK, DUSP7, CD14 |
| Insulin signaling pathway | 37 | 0.07043672 | PRKAG3, GRB2, MKNK2, HK2, RPS6KB2, PDE3B, MKNK1, HK1, PRKX, PDPK1, PPP1R3C, PRKAR2A, INS, SOS1, PIK3CA, GYS2, SHC1, CALML5, SHC4, PIK3CG, IRS2, SOCS2, BRAF, MAP2K2, PIK3CB, SOCS4, BAD, MAPK10, FBP2, IRS1, PPP1CA, TSC1, TSC2, PRKAR1A, MAPK9, RHEB, CRK |
| Aldosterone-regulated sodium reabsorption | 14 | 0.074574462 | PIK3CG, ATP1B1, IRS2, PIK3CB, NR3C2, ATP1A1, SFN, SLC9A3R2, IRS1, PDPK1, INS, PIK3CA, SCNN1G, SCNN1B |
| Cysteine and methionine metabolism | 12 | 0.085247377 | DNMT3A, CTH, AHCY, MAT1A, SDS, DNMT3L, APIP, CDO1, AMD1, TAT, SMS, DNMT3B |
| Mismatch repair | 9 | 0.09264852 | POLD3, RPA1, EXO1, RFC1, RFC2, MSH2, LIG1, PCNA, RPA4 |
| Melanoma | 21 | 0.09636964 | EGFR, PIK3CG, FGF18, FGF5, FGFR1, PDGFB, BRAF, PIK3CB, MAP2K2, MITF, TP53, FGF13, RB1, BAD, FGF21, FGF20, CDKN2A, PDGFRA, PIK3CA, PDGFD, FGF2 |

## Table 9A. Significantly enriched KEGG pathways of downregulated genes with miR-107 over-expression in HeLa cells.

| **Term** | **Count** | **PValue** | **Genes** |
| --- | --- | --- | --- |
| Neuroactive ligand-receptor interaction | 66 | 3.12E-08 | F2RL3, THRB, GABRB3, TRPV1, LEPR, GLRA3, GRIK3, GABRB1, GNRHR, VIPR2, GHRHR, GRIN2B, GRIN2C, GRIN2D, GLP2R, GHR, GABRG2, GRIN2A, NTSR1, NTSR2, GRM1, SSTR4, CRHR2, CHRM5, SSTR3, GRM7, GRM6, HTR6, F2, GPR50, FSHB, CALCR, DRD1, ADORA2A, ADCYAP1R1, NPY2R, PPYR1, FPR1, PRSS1, BDKRB2, GCGR, ADRB3, P2RY6, HRH3, P2RY4, HRH2, PRSS3, ADRA2B, GABRE, GABRA1, OPRL1, GABRA3, GRIN1, NPY1R, NPY5R, GH2, GH1, P2RX4, P2RY10, P2RX7, GPR35, GRIA2, AVPR1B, HTR2B, OPRD1, HTR2A |
| Cytokine-cytokine receptor interaction | 61 | 4.70E-06 | IL9R, ACVRL1, LEPR, IL19, TNFSF15, TNFSF14, TNFSF13, TNFSF12, CXCL12, FLT3LG, IFNG, CXCR6, CSF3R, IL15RA, GHR, EGFR, IL18RAP, TNFRSF17, TNFRSF14, IL24, CCR7, TNFRSF10B, PPBP, CCR5, TNFSF13B, CCR4, CCR3, IL12B, TNFRSF6B, CSF3, CCL1, CCL3, CSF1, CXCL9, PF4, CCL5, CCL28, LIF, CCL25, TNFRSF1B, IL12RB1, CCL23, IL23A, CCL21, IFNA5, TNFRSF19, EGF, FIGF, CSF1R, IL2RA, FLT1, CCL19, CCL16, CCL11, GH2, GH1, TSLP, TNFSF11, CXCL13, IFNA16, IL2, IL22RA2 |
| Calcium signaling pathway | 43 | 4.80E-05 | GNA14, DRD1, ADCY2, ADORA2A, PHKB, ERBB3, PPP3R2, ITPKB, BDKRB2, PRKACG, ADRB3, ATP2B3, HRH2, GRIN2C, PDE1C, GRIN2D, CAMK2D, PLCD1, CAMK2B, CALML5, EGFR, SLC8A1, PHKG2, GRIN1, GRIN2A, MYLK2, PRKCG, NTSR1, GRM1, P2RX4, CHRM5, P2RX7, ATP2A3, PLCG2, AVPR1B, HTR6, RYR1, CACNA1H, RYR2, CACNA1E, HTR2B, MYLK, HTR2A |
| Drug metabolism | 19 | 6.50E-04 | CYP3A4, GSTA3, CYP3A7, CYP2C8, GSTT2, CYP1A2, FMO4, GSTM2, CYP2A13, UGT1A9, UGT1A3, FMO1, ADH4, FMO2, UGT1A4, UGT2A1, CYP2A6, GSTZ1, UGT2B10, UGT2B15, UGT2B28 |
| B cell receptor signaling pathway | 21 | 0.001097564 | PIK3CG, BCL10, PPP3R2, VAV2, VAV1, CARD11, DAPP1, FCGR2B, RAC2, FCGR2C, LILRB3, SOS2, PLCG2, RAC1, CD79B, NFATC4, CD79A, INPP5D, NFATC2, AKT3, NFATC1, AKT2 |
| Chemokine signaling pathway | 40 | 0.00159567 | CCL1, CCL3, ADCY2, FGR, GNAI1, CXCL9, PF4, CCL5, CXCL12, CCL28, PRKACG, CCL25, CCL23, RAC2, CCL21, SOS2, RAC1, CXCR6, GNG3, GNG4, AKT3, GNG7, AKT2, PIK3CG, ITK, CCL19, VAV2, CCL16, VAV1, CCL11, GNGT1, CCR7, PPBP, CCR5, ARRB2, CCR4, CXCL13, CCR3, GRK7, JAK3 |
| Amyotrophic lateral sclerosis (ALS) | 16 | 0.002381652 | BID, MAP2K3, GRIN1, TP53, GRIN2A, PPP3R2, GPX1, TNFRSF1B, SLC1A2, GRIA2, GRIN2B, GRIN2C, GRIN2D, BCL2, RAC1, NEFL |
| Systemic lupus erythematosus | 24 | 0.003523246 | HIST1H2AB, C7, LOC340096, HIST4H4, HIST1H2BN, HIST1H4A, GRIN2B, HIST1H2BL, IFNG, C2, HIST1H4D, HIST3H2BB, HIST1H4G, HIST1H4H, HIST1H2BA, HIST1H3J, HIST1H2BC, C4A, HIST1H2BF, GRIN2A, ACTN2, HLA-DQA2, HIST2H3C, C8A, C1QB, CD86, CD80, FCGR2B, FCGR2C, HIST1H3A, FCGR2A, HIST1H3E, HIST1H3G, HIST1H3H |
| Toll-like receptor signaling pathway | 24 | 0.004595675 | PIK3CG, CCL3, MAP2K3, TIRAP, CXCL9, MAPK10, TLR5, TLR6, CCL5, TLR7, TLR9, CD86, CD80, IRF5, IRF7, IFNA5, RAC1, CASP8, IL12B, IFNA16, AKT3, TRAF3, AKT2, SPP1 |
| Focal adhesion | 40 | 0.006035583 | MYL2, PAK6, LAMB4, RAC2, ITGB8, PAK3, BCL2, SOS2, ITGB6, RAC1, COL6A2, COL6A1, TNN, EGF, FIGF, AKT3, SPP1, THBS4, AKT2, PIK3CG, EGFR, TNXB, FLT1, MYLK2, PRKCG, ACTN2, ITGA3, MAPK10, BIRC3, VAV2, COL5A3, VAV1, COL4A6, LAMA2, RASGRF1, ITGA8, ITGA7, GRLF1, LAMC2, MYLK |
| Drug metabolism | 13 | 0.007147657 | CYP3A4, CYP3A7, NAT2, UPP2, TPMT, CYP2A13, UMPS, UGT1A9, UGT1A3, UGT1A4, CYP2A6, UGT2A1, UGT2B10, UGT2B15, UGT2B28 |
| Arrhythmogenic right ventricular cardiomyopathy (ARVC) | 19 | 0.007550533 | SLC8A1, CACNG7, CACNG5, CACNB1, LEF1, CACNB2, ITGA3, ACTN2, CACNA2D2, TCF7L1, LAMA2, JUP, DES, ITGB8, ITGA8, DMD, ITGA7, ITGB6, RYR2 |
| Steroid hormone biosynthesis | 13 | 0.01256381 | CYP3A4, CYP3A7, CYP11A1, HSD17B2, SULT2B1, CYP17A1, UGT1A9, UGT1A3, UGT1A4, UGT2A1, HSD17B3, UGT2B10, SULT1E1, UGT2B15, UGT2B28 |
| Axon guidance | 27 | 0.014162386 | ABLIM2, PLXNC1, NRP1, GNAI1, EFNA3, PPP3R2, EPHB3, CXCL12, EPHB1, PAK6, SEMA5B, RAC2, PAK3, ROBO1, RAC1, SEMA3D, NFATC4, NFATC2, RASA1, NFATC1, LIMK1, EFNB1, PLXNB3, ARHGEF12, SEMA6B, RGS3, EFNA5 |
| Jak-STAT signaling pathway | 31 | 0.015480263 | CSF3, IL9R, LEPR, IL19, SPRY4, LIF, SPRY1, IL12RB1, IL23A, IFNA5, SOS2, IFNG, CSF3R, IL15RA, AKT3, GHR, AKT2, PIK3CG, IL2RA, SOCS3, SOCS5, IL24, CISH, GH2, GH1, TSLP, IL12B, JAK3, IFNA16, IL2, IL22RA2 |
| MAPK signaling pathway | 48 | 0.017179618 | FGF19, FGF18, FGF17, DUSP10, CACNB1, CACNB2, PPP3R2, PRKACG, BDNF, MAP3K3, RAC2, RASGRP4, ELK4, SOS2, RAC1, PLA2G1B, HSPA6, NFATC4, EGF, NFATC2, FGF1, RASA1, AKT3, AKT2, PTPN7, EGFR, PLA2G10, CACNG7, MAP2K3, PTPN5, CACNG5, TP53, PRKCG, FGF22, MAPK10, CACNA2D2, CDC25B, MAP4K4, ARRB2, RASGRF1, MAPK8IP2, CACNA1H, MAPK8IP3, CACNA1E, PLA2G3, DUSP7, MAP3K12, PLA2G2F |
| Wnt signaling pathway | 30 | 0.018927024 | CER1, PPARD, WNT16, PPP3R2, TCF7L1, PRKACG, WNT1, CSNK2A1, RAC2, RAC1, CAMK2D, NFATC4, CAMK2B, NFATC2, FOSL1, WNT8A, NFATC1, WNT10A, VANGL2, TP53, LEF1, PRKCG, MAPK10, DKK4, WNT2B, FZD10, PRICKLE2, WNT9B, WIF1, WNT7A |
| Tight junction | 27 | 0.02250534 | CLDN7, RAB3B, MYL2, VAPA, GNAI1, CLDN6, AMOTL1, CLDN14, LLGL1, CSNK2A1, PPP2R2B, AKT3, AKT2, F11R, SYMPK, PARD6B, INADL, HCLS1, MYH3, MYH2, PRKCG, ACTN2, TJP1, EPB41L1, MYH11, MYH13, JAM3 |
| Linoleic acid metabolism | 9 | 0.022809506 | CYP3A4, CYP2J2, CYP3A7, PLA2G10, CYP2C8, PLA2G1B, CYP1A2, PLA2G3, PLA2G2F |
| T cell receptor signaling pathway | 22 | 0.036961471 | PIK3CG, ITK, BCL10, CD8A, PPP3R2, VAV2, VAV1, PDCD1, PAK6, CARD11, PAK3, SOS2, IFNG, ZAP70, NFATC4, GRAP2, NFATC2, AKT3, TEC, IL2, NFATC1, AKT2 |
| Pathways in cancer | 55 | 0.037712027 | FGF19, FGF18, PPARD, FGF17, MMP9, MITF, MMP1, FLT3LG, WNT1, CASP8, PAX8, CSF3R, FGF1, AKT3, AKT2, EGFR, PIK3CG, WNT10A, RET, TP53, LEF1, PRKCG, FGF22, RAD51, JUP, SMO, WNT9B, MDM2, LAMC2, BID, WNT16, EGLN2, TCF7L1, LAMB4, RAC2, BCL2, SOS2, RAC1, EGF, FIGF, TRAF5, WNT8A, TRAF3, CSF1R, KLK3, ITGA3, MAPK10, BIRC3, COL4A6, WNT2B, LAMA2, FZD10, PLCG2, PTCH2, WNT7A |
| ECM-receptor interaction | 18 | 0.040691332 | TNXB, ITGA3, COL5A3, COL4A6, LAMA2, LAMB4, GP1BB, ITGB8, ITGA8, ITGA7, ITGB6, COL6A2, COL6A1, LAMC2, TNN, SV2A, SPP1, THBS4 |
| Fc epsilon RI signaling pathway | 17 | 0.041389111 | PIK3CG, PLA2G10, MAP2K3, MAPK10, VAV2, VAV1, GAB2, RAC2, SOS2, PLCG2, RAC1, PLA2G1B, INPP5D, PLA2G3, AKT3, AKT2, PLA2G2F |
| Retinol metabolism | 13 | 0.041959781 | CYP3A4, CYP3A7, CYP2C8, DHRS9, CYP1A2, CYP2A13, UGT1A9, UGT1A3, ADH4, UGT1A4, CYP2A6, UGT2A1, UGT2B10, UGT2B15, UGT2B28 |
| Metabolism of xenobiotics by cytochrome P450 | 14 | 0.042287129 | CYP3A4, GSTA3, CYP3A7, CYP2C8, GSTT2, CYP1A2, GSTM2, UGT1A9, UGT1A3, ADH4, UGT1A4, GSTZ1, UGT2A1, UGT2B10, UGT2B15, UGT2B28 |
| Hypertrophic cardiomyopathy (HCM) | 18 | 0.044959842 | SLC8A1, MYL2, CACNG7, MYBPC3, CACNG5, CACNB1, CACNB2, ITGA3, CACNA2D2, LAMA2, ACE, DES, ITGB8, ITGA8, DMD, ITGA7, ITGB6, RYR2 |
| Intestinal immune network for IgA production | 12 | 0.047075632 | TNFRSF17, TNFSF13, PIGR, TNFSF12, CXCL12, HLA-DQA2, CCL28, CCL25, CD86, CD80, TNFSF13B, IL15RA, IL2 |
| Basal cell carcinoma | 13 | 0.047578143 | WNT10A, WNT16, TP53, LEF1, TCF7L1, WNT2B, WNT1, SMO, FZD10, WNT9B, PTCH2, WNT7A, WNT8A |
| Dilated cardiomyopathy | 19 | 0.048243594 | SLC8A1, ADCY2, MYL2, CACNG7, MYBPC3, CACNG5, CACNB1, CACNB2, ITGA3, CACNA2D2, LAMA2, PRKACG, DES, ITGB8, ITGA8, DMD, ITGA7, ITGB6, RYR2 |
| Hematopoietic cell lineage | 18 | 0.049538778 | CSF3, CR1, IL9R, IL2RA, CD8A, CSF1, ANPEP, ITGA3, CD1E, FLT3LG, CD1D, GP1BB, CD33, MS4A1, CSF3R, CD5, CSF1R, CD7 |
| Leukocyte transendothelial migration | 23 | 0.050291969 | PIK3CG, F11R, CLDN7, ITGAL, ITK, MYL2, GNAI1, CLDN6, MMP9, NOX1, PRKCG, ACTN2, VAV2, VAV1, CXCL12, CLDN14, VCAM1, CYBB, RAC2, RAC1, PLCG2, GRLF1, JAM3 |
| Melanogenesis | 20 | 0.051257445 | WNT10A, WNT16, ADCY2, GNAO1, GNAI1, MITF, LEF1, PRKCG, POMC, TCF7L1, WNT2B, PRKACG, WNT1, FZD10, WNT9B, CAMK2D, CAMK2B, CALML5, WNT7A, WNT8A |
| Caffeine metabolism | 4 | 0.051524115 | CYP2A13, NAT2, CYP2A6, CYP1A2 |
| ErbB signaling pathway | 18 | 0.054437361 | EGFR, PIK3CG, ERBB3, BTC, PRKCG, MAPK10, PAK6, EREG, PAK3, PLCG2, SOS2, CAMK2D, CAMK2B, NRG1, EGF, ABL2, AKT3, AKT2 |
| Complement and coagulation cascades | 15 | 0.058572599 | C7, CR1, C4A, MASP1, BDKRB2, C8A, C1QB, FGG, THBD, F5, FGB, F2, SERPINC1, SERPIND1, C2 |
| Viral myocarditis | 15 | 0.071566001 | BID, ITGAL, MYH3, MYH2, HLA-DQA2, LAMA2, CD86, CD80, RAC2, DMD, CASP8, RAC1, MYH11, MYH13, ABL2 |
| Pentose and glucuronate interconversions | 6 | 0.074677782 | UGT1A9, UGT1A3, UGT1A4, UGT2A1, UGT2B10, UGT2B15, UGT2B28, XYLB |
| Ether lipid metabolism | 9 | 0.076582051 | PLA2G10, ENPP2, PPAP2C, PAFAH2, PLA2G1B, PPAP2A, PLA2G3, PPAP2B, PLA2G2F |
| Natural killer cell mediated cytotoxicity | 24 | 0.092419276 | BID, PIK3CG, ITGAL, PPP3R2, PRKCG, VAV2, VAV1, CD48, TNFRSF10B, RAC2, ULBP1, IFNA5, SOS2, PLCG2, IFNG, RAC1, ZAP70, NFATC4, NFATC2, IFNA16, KIR2DL2, KLRC1, KIR2DL4, NFATC1 |
| Dorso-ventral axis formation | 7 | 0.097085991 | NOTCH3, EGFR, NOTCH2, ETV7, HSD3B7, SOS2, NOTCH4, PIWIL2 |
| Keratan sulfate biosynthesis | 5 | 0.098493657 | B4GALT2, CHST6, B3GNT7, CHST2, CHST4 |
| Androgen and estrogen metabolism | 9 | 0.099866888 | UGT1A9, HSD17B2, UGT1A3, UGT1A4, SULT2B1, UGT2A1, HSD17B3, UGT2B10, SULT1E1, UGT2B15, UGT2B28 |

## Table 9B. Significantly enriched KEGG pathways of upregulated genes with miR-107 inhibition in HeLa cells.

| **Term** | **Count** | **PValue** | **Genes** |
| --- | --- | --- | --- |
| Olfactory transduction | 83 | 3.72E-05 | OR2AK2, OR7A17, OR51D1, OR1A2, OR1E2, OR1N2, OR11A1, OR51Q1, CNGB1, OR52D1, OR4C3, OR2V2, OR6C2, OR6C3, OR2T11, OR2T12, OR7C1, OR5B21, OR5I1, CLCA1, OR5M1, OR52B6, OR4A47, OR4F15, OR5M9, OR10AG1, OR4D6, OR4K15, OR8J1, OR4K14, OR2D3, OR2L8, OR8U9, OR6F1, OR2T1, OR1L8, OR2T4, OR8H3, OR2T3, OR51F2, OR5A1, OR10G2, OR4F4, OR2L13, OR10G4, OR2G2, OR56B4, OR52N1, OR5AP2, OR5B12, OR6Y1, OR5F1, OR10R2, CALML5, OR13C8, OR51V1, OR5P3, OR2A5, OR7G1, OR2AG2, OR2A14, OR2A7, OR5M11, OR8G5, OR11H4, OR1F1, OR2M4, OR10H3, OR56A3, OR10Z1, OR51M1, OR12D2, OR52M1, OR2T27, OR3A1, OR52E8, OR52W1, PDC, OR6K3, OR51I1, OR10V1, OR51A7, OR6X1 |
| Arrhythmogenic right ventricular cardiomyopathy (ARVC) | 18 | 0.036986407 | ACTB, TCF7, CACNA2D1, CACNG7, CACNB1, CACNB2, ITGA4, ITGB1, CTNNA3, CTNNB1, LAMA2, ACTG1, JUP, ATP2A2, ITGB8, ITGAV, ITGB7, SGCA |
| Non-small cell lung cancer | 14 | 0.037310827 | EGFR, PIK3CB, MAP2K2, RXRG, TP53, PRKCG, RB1, BAD, PDPK1, RASSF5, CDKN2A, RASSF1, PIK3R1, AKT2 |
| Intestinal immune network for IgA production | 13 | 0.039393044 | TNFRSF13C, TNFSF13, PIGR, ITGA4, TNFSF12, CCL28, TGFB2, CCR9, CCL25, TNFSF13B, CD80, CXCR4, ITGB7, MAP3K14 |
| Melanoma | 17 | 0.03967277 | EGFR, FGF5, FGF8, PDGFB, PIK3CB, MAP2K2, FGF14, MITF, TP53, FGF13, RB1, BAD, FGF20, CDKN2A, FGF2, PIK3R1, AKT2 |
| Prostate cancer | 20 | 0.044081768 | EGFR, AR, TCF7, HSP90AA1, PDGFB, PIK3CB, MAP2K2, RELA, TP53, RB1, BAD, CTNNB1, CCNE2, PDPK1, INS, BCL2, CREB3L1, CREB3L3, PIK3R1, AKT2 |
| Fc gamma R-mediated phagocytosis | 21 | 0.045081949 | DNM3, PTPRC, PPAP2C, NCF1, PIK3CB, HCK, WASF1, SPHK1, RPS6KB2, PRKCG, ARPC4, VASP, FCGR2B, ARPC2, GSN, PLA2G4F, PPAP2A, PIK3R1, PLA2G4E, AKT2, DNM2 |
| Hypertrophic cardiomyopathy (HCM) | 19 | 0.052660167 | ACTB, CACNA2D1, CACNG7, CACNB1, CACNB2, MYH7, MYH6, ITGA4, ITGB1, TPM4, TGFB2, LAMA2, ACTG1, ACE, ATP2A2, ITGB8, ITGAV, ITGB7, SGCA |
| Dilated cardiomyopathy | 20 | 0.059011294 | ACTB, ADCY4, CACNA2D1, ADCY1, CACNG7, CACNB1, CACNB2, MYH7, MYH6, ITGA4, ITGB1, TPM4, TGFB2, LAMA2, ACTG1, ATP2A2, ITGB8, ITGAV, ITGB7, SGCA |
| Endometrial cancer | 13 | 0.059127404 | EGFR, TCF7, PIK3CB, MAP2K2, TP53, BAD, CTNNA3, CTNNB1, PDPK1, ILK, PIK3R1, AKT2, AXIN1 |
| Pathways in cancer | 57 | 0.082423901 | FGF5, FGF8, PDGFB, FGF14, MITF, SPI1, FGF13, TGFB2, CTNNB1, CCNE2, CASP3, CDKN2A, CASP8, PAX8, CSF3R, RARA, FGF2, AKT2, EGFR, AR, HSP90AA1, CTBP2, PIK3CB, RELA, RXRG, TP53, PRKCG, RB1, FGF20, CTNNA3, RAD51, DAPK1, JUP, HIF1A, WNT16, KITLG, ITGB1, ITGAV, BCL2, PIK3R1, TRAF4, AXIN1, CEBPA, TCF7, MAP2K2, BRCA2, FZD3, MAPK10, BAD, COL4A6, FZD7, DVL1, WNT2B, LAMA2, RASSF5, RASSF1, JAK1 |
| Renin-angiotensin system | 6 | 0.083398828 | LNPEP, ACE, AGTR2, MAS1, MME, NLN |
| Sphingolipid metabolism | 10 | 0.094687835 | SPTLC1, PPAP2C, SPHK1, ARSA, CERK, B4GALT6, PPAP2A, ASAH1, SMPD3, GBA |
| Drug metabolism | 14 | 0.096290904 | CYP3A5, CYP2C8, ADH5, CYP1A2, GSTM5, ALDH3B1, GSTM1, UGT1A7, UGT1A6, UGT2B17, UGT1A9, GSTM4, FMO1, ALDH1A3, FMO2, UGT1A5, UGT2B4 |

## Table 10A. Enriched KEGG pathways of genes differentially expressed with both miR-107 over-expression and inhibition in HEK-293 cells.

| **Term** | **Count** | **PValue** | **Genes** |
| --- | --- | --- | --- |
| Neuroactive ligand-receptor interaction | 20 | 1.22E-04 | OPRM1, PTGER1, GRIK1, GABRB3, LEPR, GABRA5, GRIK4, LEP, EDNRB, P2RX7, GRIN2B, CHRM2, GRM7, HTR7, MC2R, GRM6, TBXA2R, MAS1, GABRQ, HTR1E |
| MAPK signaling pathway | 17 | 0.004119014 | FGF5, IL1R1, PDGFB, CACNG5, DUSP10, CACNB1, MAPK11, MAPK10, DUSP4, BDNF, MAP3K4, MAPT, NTRK1, PDGFRA, CACNA1E, PLA2G2E, DUSP7 |
| Cytokine-cytokine receptor interaction | 14 | 0.038695019 | CCL3, IL1R1, PDGFB, CXCL5, LEPR, CSF1, CCL19, KITLG, TNFRSF8, CCL27, LEP, CCL25, CCR3, PDGFRA |
| Systemic lupus erythematosus | 7 | 0.06586395 | HIST1H2BA, GRIN2B, FCGR2B, HIST1H2BF, FCGR2A, HIST3H3, HLA-DQA2 |
| Calcium signaling pathway | 10 | 0.067565625 | PTGER1, EDNRB, P2RX7, CHRM2, HTR7, PDGFRA, TBXA2R, CACNA1E, CALML5, MYLK |
| Fructose and mannose metabolism | 4 | 0.073827638 | PFKFB3, GMPPA, HK1, FBP2 |
| Cell adhesion molecules (CAMs) | 8 | 0.086637897 | PVRL1, NFASC, CNTN1, ITGB2, NLGN3, CD4, ITGA4, HLA-DQA2 |

## Table 10B. Enriched KEGG pathways of genes differentially expressed with both miR-107 over-expression and inhibition in HeLa cells.

| **Term** | **Count** | **PValue** | **Genes** |
| --- | --- | --- | --- |
| Intestinal immune network for IgA production | 6 | 0.008874811 | CCL25, TNFSF13B, CD80, TNFSF13, TNFSF12, PIGR, CCL28 |
| Focal adhesion | 12 | 0.016340087 | EGFR, LAMA2, ITGB8, BCL2, GRLF1, PRKCG, TNN, MAPK10, COL4A6, MYLK, THBS4, AKT2 |
| Neuroactive ligand-receptor interaction | 14 | 0.016677475 | F2RL3, GABRA3, PPYR1, GRIN1, GNRHR, ADRB3, GH1, CHRM5, P2RX7, P2RY6, F2, GRM6, GPR50, OPRD1 |
| Pathways in cancer | 16 | 0.024805825 | EGFR, WNT16, MITF, TP53, PRKCG, MAPK10, COL4A6, WNT2B, RAD51, LAMA2, JUP, BCL2, PAX8, CASP8, CSF3R, AKT2 |
| Linoleic acid metabolism | 4 | 0.036257941 | CYP2J2, PLA2G10, CYP2C8, CYP1A2 |
| Cytokine-cytokine receptor interaction | 13 | 0.042358889 | EGFR, CSF1, CCL19, TNFSF13, TNFSF12, CCL5, CCL28, CCL25, GH1, IL12RB1, TNFSF13B, CCL21, CSF3R, IL12B |
| GnRH signaling pathway | 7 | 0.043767788 | EGFR, PLA2G10, MAP3K3, MAP2K3, GNRHR, MAPK10, CALML5 |
| MAPK signaling pathway | 13 | 0.047786581 | EGFR, PLA2G10, MAP2K3, CACNG7, TP53, DUSP10, CACNB1, CACNB2, PRKCG, MAPK10, MAP3K3, DUSP7, AKT2 |
| Arrhythmogenic right ventricular cardiomyopathy (ARVC) | 6 | 0.049149291 | JUP, LAMA2, ITGB8, CACNG7, CACNB1, CACNB2 |
| Toll-like receptor signaling pathway | 7 | 0.049478017 | CD80, MAP2K3, CASP8, MAPK10, IL12B, CCL5, AKT2 |
| Amyotrophic lateral sclerosis (ALS) | 5 | 0.050003636 | BCL2, MAP2K3, GRIN1, TP53, NEFL |
| Arachidonic acid metabolism | 5 | 0.059099028 | CYP2J2, PLA2G10, GPX5, CYP2C8, PTGS1 |
| Ether lipid metabolism | 4 | 0.063491526 | PLA2G10, ENPP2, PPAP2C, PPAP2A |
| Hypertrophic cardiomyopathy (HCM) | 6 | 0.072593314 | LAMA2, ACE, ITGB8, CACNG7, CACNB1, CACNB2 |
| Drug metabolism | 5 | 0.079739976 | UGT1A9, FMO1, CYP2C8, FMO2, CYP1A2 |
| Glioma | 5 | 0.083488687 | EGFR, TP53, PRKCG, CALML5, AKT2 |
| Calcium signaling pathway | 9 | 0.091879438 | EGFR, ADRB3, P2RX7, CHRM5, PHKB, GRIN1, PRKCG, CALML5, MYLK |
| Type I diabetes mellitus | 4 | 0.097739024 | ICA1, CD80, PTPRN2, IL12B |

## Figure 10C. Enriched KEGG pathways of genes modulated with either miR-107 over-expression or inhibition across both HEK-293 and HeLa cell models.

| **Term** | **Count** | **PValue** | **Genes** |
| --- | --- | --- | --- |
| Arrhythmogenic right ventricular cardiomyopathy (ARVC) | 20 | 0.010340073 | ACTB, TCF7, CACNG7, CACNG5, CACNB1, CACNB2, ACTN2, ITGA4, ITGB1, CTNNA3, CTNNB1, LAMA2, JUP, ACTG1, ATP2A2, ITGB8, DMD, ITGAV, ITGA7, SGCA |
| Melanoma | 19 | 0.010683996 | EGFR, PIK3CG, FGF18, FGF5, FGF8, PDGFB, PIK3CB, MAP2K2, MITF, TP53, FGF13, RB1, BAD, FGF20, CDKN2A, FGF1, EGF, FGF2, AKT3 |
| Systemic lupus erythematosus | 23 | 0.024038798 | HIST1H2AC, C7, LOC340096, HIST1H4L, C6, C1R, HIST1H2BN, HIST1H4A, GRIN2B, HIST1H4B, H2AFY, C2, HIST1H4I, HIST3H2BB, HIST1H4G, HIST1H2BA, HIST1H2BC, C4A, HIST1H2BF, ACTN2, HLA-DQA2, HIST2H3C, C8A, CD86, CD80, FCGR2B, FCGR2C, HIST1H3A, HIST1H3B, FCGR2A, HIST1H3G, HIST1H3H |
| Tight junction | 29 | 0.026233729 | VAPA, GNAI1, CLDN6, MRAS, AMOTL1, CTNNB1, ACTG1, CTTN, CSNK2A1, PPP2CB, PPP2R2B, AKT3, ACTB, PARD6B, INADL, EPB41, HCLS1, CRB3, ACTN2, MYH7, MYH6, CLDN20, CTNNA3, TJP1, EPB41L1, MYH11, CLDN2, MYH13, TJP2 |
| Prostate cancer | 21 | 0.027297134 | EGFR, PIK3CG, AR, TCF7, HSP90AA1, PDGFB, PIK3CB, MAP2K2, RELA, TP53, CREB5, RB1, BAD, CTNNB1, CCNE2, PDPK1, INS, CREB3L1, CREB3L3, EGF, AKT3 |
| Pathways in cancer | 61 | 0.028792915 | FGF18, FGF5, FGF8, PDGFB, MMP9, MITF, FGF13, MMP1, TGFB2, CTNNB1, CCNE2, WNT1, CDKN2A, CASP8, PAX8, FGF1, FGF2, AKT3, EGFR, PIK3CG, WNT10A, AR, HSP90AA1, CTBP2, PIK3CB, RELA, TP53, RB1, FGF20, CTNNA3, DAPK1, RAD51, JUP, HIF1A, LAMC2, WNT16, KITLG, EGLN2, ITGB1, LAMB4, RAC2, ITGAV, EGF, TRAF5, TRAF4, AXIN1, CSF1R, CEBPA, TCF7, MAP2K2, BRCA2, BAD, MAPK10, COL4A6, FZD7, DVL1, LAMA2, RASSF5, FZD10, RASSF1, JAK1 |
| MAPK signaling pathway | 51 | 0.029764192 | FGF18, FGF5, FGF8, PDGFB, FGF13, TGFB2, BDNF, MAP3K4, MAPT, IL1B, FGF1, FGF2, MAP2K6, AKT3, EGFR, RELA, CACNG7, CACNG5, TP53, FGF20, FLNA, MAP4K3, MAP4K4, RASGRF1, PLA2G2F, MRAS, DUSP10, CACNB1, PPM1A, MKNK2, MKNK1, CACNB2, GNG12, PPM1B, RASGRP3, RAC2, MAP3K3, NFATC4, EGF, PLA2G10, MAP2K2, MAP2K3, NR4A1, MAPK11, MAPK10, DUSP4, MAPK8IP2, CACNA1H, MAPK8IP3, CACNA1E, DUSP7 |
| Glycerophospholipid metabolism | 17 | 0.030725902 | GPD2, ACHE, PLA2G10, PPAP2C, LYPLA2, LYPLA1, DGKA, AGPAT6, DGKG, PEMT, PHOSPHO1, PPAP2A, PPAP2B, AGPAT3, CHAT, AGPAT2, PLA2G2F |
| Hypertrophic cardiomyopathy (HCM) | 20 | 0.032541801 | ACTB, CACNG7, CACNG5, CACNB1, CACNB2, MYH7, MYH6, ITGA4, ITGB1, TPM4, TGFB2, LAMA2, ACTG1, ACE, ATP2A2, ITGB8, DMD, ITGAV, ITGA7, SGCA |
| Keratan sulfate biosynthesis | 6 | 0.040812052 | B4GALT2, CHST6, B3GNT7, CHST2, B3GNT1, CHST4 |
| Intestinal immune network for IgA production | 13 | 0.043168707 | TNFRSF13C, TNFSF13, PIGR, ITGA4, TNFSF12, CXCL12, CCL28, HLA-DQA2, TGFB2, CCL25, CD86, CD80, CXCR4, IL15RA |
| Viral myocarditis | 17 | 0.04422688 | ACTB, MYH7, HLA-C, MYH6, HLA-DQA2, LAMA2, ACTG1, CD86, CD80, RAC2, FYN, DMD, CASP8, MYH11, MYH13, ABL2, SGCA |
| Fructose and mannose metabolism | 10 | 0.048170794 | KHK, GMPPB, ALDOA, MTMR1, SORD, PFKFB3, GMPPA, HK2, HK1, TNNI3K, FPGT |
| Fc gamma R-mediated phagocytosis | 21 | 0.05090506 | PIK3CG, PTPRC, LIMK1, PPAP2C, NCF1, PIK3CB, HCK, WASF1, RPS6KB2, ARPC4, GAB2, FCGR2B, RAC2, ARPC2, FCGR2C, GSN, FCGR2A, INPP5D, PPAP2A, PPAP2B, AKT3, DNM2 |
| Regulation of actin cytoskeleton | 41 | 0.052760665 | GNA13, FGF18, FGF5, FGF8, PDGFB, MRAS, WASF1, ARPC4, FGF13, BDKRB2, GNG12, ITGB1, ACTG1, PFN1, PFN2, RAC2, INS, ITGB8, ARPC2, GSN, ITGAV, EGF, FGF1, FGF2, ACTB, PIK3CG, EGFR, ARHGEF1, LIMK1, MAP2K2, PIK3CB, BAIAP2, MYLK2, ACTN2, ITGA4, FGF20, PPP1CA, CHRM2, ITGA7, GRLF1, MYLK |
| Primary immunodeficiency | 10 | 0.05685415 | PTPRC, CD8A, TAP2, ZAP70, TNFRSF13C, IL2RG, CD4, CD79A, JAK3, RFXAP |
| Ether lipid metabolism | 10 | 0.05685415 | AGPAT6, PLA2G10, ENPP2, PPAP2C, PAFAH2, PPAP2A, AGPAT3, PPAP2B, AGPAT2, PLA2G2F |
| Endometrial cancer | 13 | 0.064441209 | PIK3CG, EGFR, TCF7, PIK3CB, MAP2K2, TP53, BAD, CTNNA3, CTNNB1, PDPK1, EGF, AKT3, AXIN1 |
| Hematopoietic cell lineage | 19 | 0.064831216 | CSF3, CR1, IL9R, CD3G, CD8A, CSF1, KITLG, MME, ANPEP, IL6R, ITGA4, GP9, CD37, CD44, CD34, CD33, IL1B, CD4, CSF1R |
| Neuroactive ligand-receptor interaction | 47 | 0.065923043 | OPRM1, DRD1, THRA, GABRB3, DRD2, LEPR, GABRB1, PPYR1, FPR1, PRSS1, GNRHR, BDKRB2, VIPR2, GHRHR, ADRB3, HCRTR1, P2RY6, GRIN2B, HRH2, PRSS3, MAS1, ADRA2C, CALCRL, GLP2R, GABRQ, GPR156, PTGER1, GABRG2, PTGER3, GRIN1, NPY5R, P2RX5, GH1, GPR35, P2RX7, P2RY10, P2RX1, GRIA2, CHRM2, GRIA1, GRM7, GRM6, HTR6, MC4R, GPR50, GHSR, HTR2A |
| Dilated cardiomyopathy | 20 | 0.066051542 | ACTB, ADCY1, CACNG7, CACNG5, CACNB1, CACNB2, MYH7, MYH6, ITGA4, ITGB1, TPM4, TGFB2, LAMA2, ACTG1, ATP2A2, ITGB8, DMD, ITGAV, ITGA7, SGCA |
| Non-small cell lung cancer | 13 | 0.081901372 | PIK3CG, EGFR, PIK3CB, MAP2K2, TP53, BAD, RB1, PDPK1, RASSF5, CDKN2A, RASSF1, EGF, AKT3 |
| Renin-angiotensin system | 6 | 0.087442389 | LNPEP, ACE, MAS1, MME, CPA3, ANPEP |
| Pancreatic cancer | 16 | 0.089470143 | EGFR, PIK3CG, PIK3CB, RELA, TP53, BRCA2, RB1, MAPK10, BAD, TGFB2, RAD51, CDKN2A, RAC2, JAK1, EGF, AKT3 |
