# Supplementary figures and images for: Alternative mRNA fates identified in microRNA-associated transcriptome analysis
Source: BMC Genomics. 2012 Oct 19;13:561. doi: 10.1186/1471-2164-13-561 (PMC3505728; doi:10.1186/1471-2164-13-561)

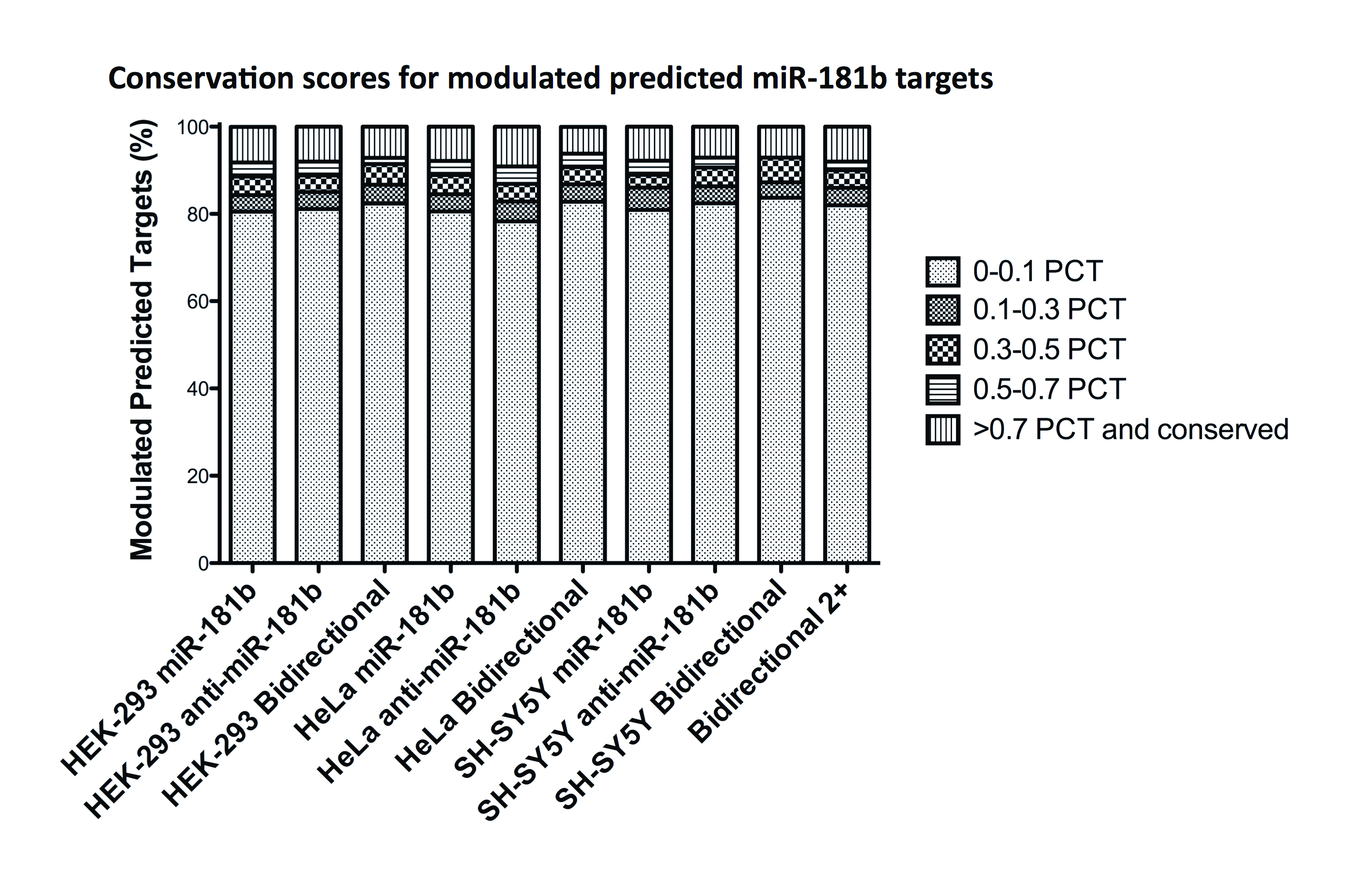

Supplement: Additional file 2 — Figure S1. Conservation scores for modulated predicted miR-181b targets, as predicted using Targetscan. PCT: Probability of conserved targeting; the lower the probabilistic value, the poorer the conservation of the predicted binding site across multiple species. Bidirectional 2+ indicates genes modulated by both miR-181b over-expression and inhibition across two or more cell models. [file 1471-2164-13-561-S2.tiff]

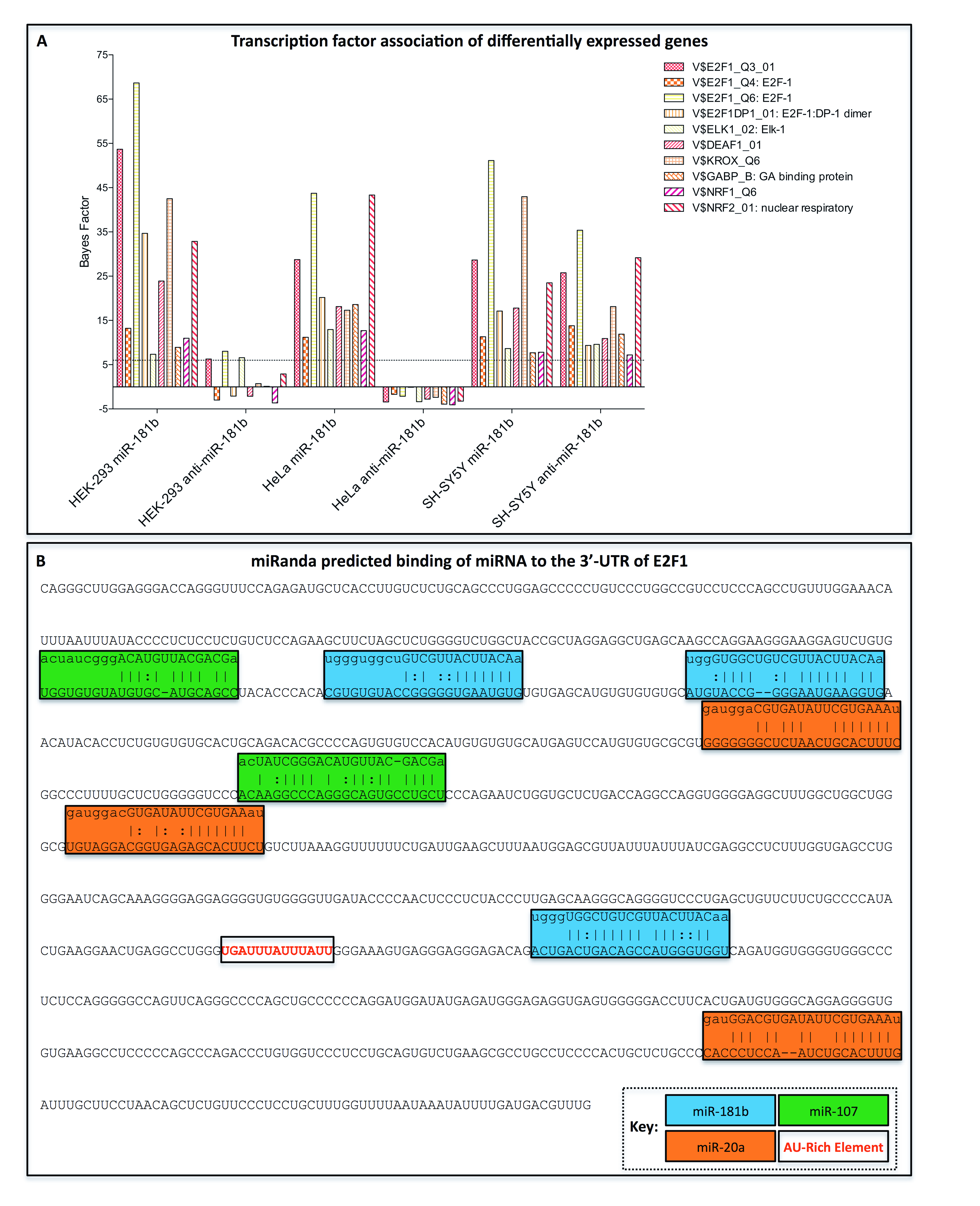

Supplement: Additional file 3 — Figure S2. Bioinformatic evidence for a role of E2F1 transcription factor in contributing to miRNA-associated expression profiles. Panel A graphically represents the Transcription factor association of canonically modulated genes subsequent miR-181b over-expression or inhibition in HEK-293, HeLa, and SH-SY5Y cell models. The TRANSFAC function of GATHER was used to identify significantly enriched transcription factor signatures within modulated genes. A Bayes factor of 6 was used for threshold significance, which in each case corresponded to p<0.0001. Panel B illustrates predicted binding sites for schizophrenia-associated miR-181b, miR-107, and miR-20a in the 3′-UTR of E2F1, as well as the AU-rich element in this 3′-UTR. These predicted binding sites were identified using the miRanda shell algorithm; only sites with threshold scores greater than 120 are shown in this figure. [file 1471-2164-13-561-S3.tiff]

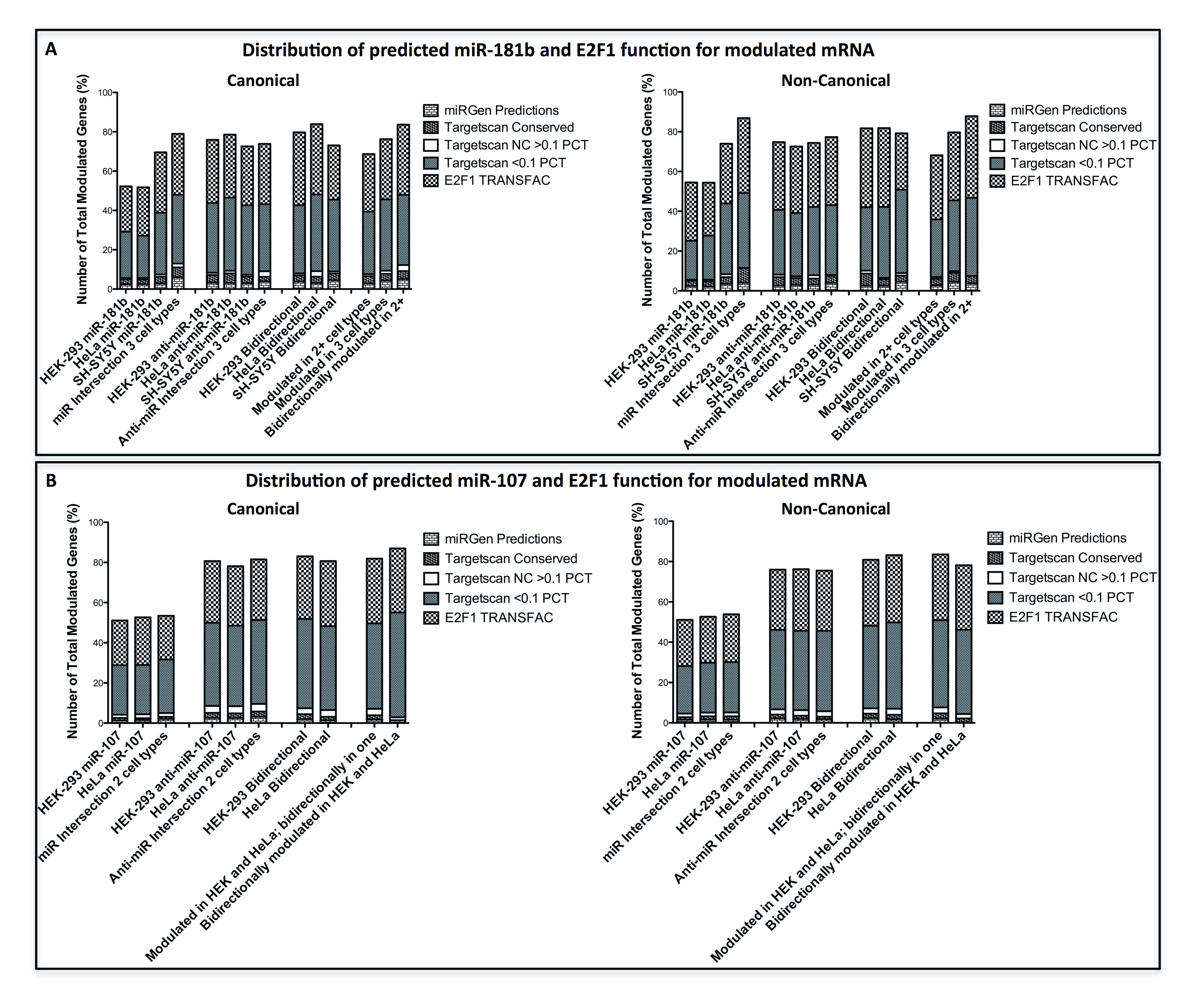

Supplement: Additional file 4 — Figure S3. Distribution of predicted miRNA and E2F1 function for modulated mRNA. Panel A shows the proportion of modulated genes that can be attributed to predicted miR-181b and E2F1 function across individual and multiple cell models; whilst Panel B shows this for miR-107 predicted function. In both panels data is presented for canonical (negative miRNA-mRNA correlation) and non-canonical (positive miRNA-mRNA correlation) patterns of correlation. Differentially expressed genes are classified as being predicted in preferential fashion by: Targetscan conserved predicted miR-181b target; Targetscan non-conserved predicted miR-181b target, with PCT (probability of conservation) scores <0.1; Targetscan non-conserved predicted miR-181b target, with PCT scores >0.1; not predicted as a miR-181b target by Targetscan, but predicted by the miRGen algorithm; predicted as containing E2F1 recognition signatures using the TRANSFAC algorithm. [file 1471-2164-13-561-S4.tiff]

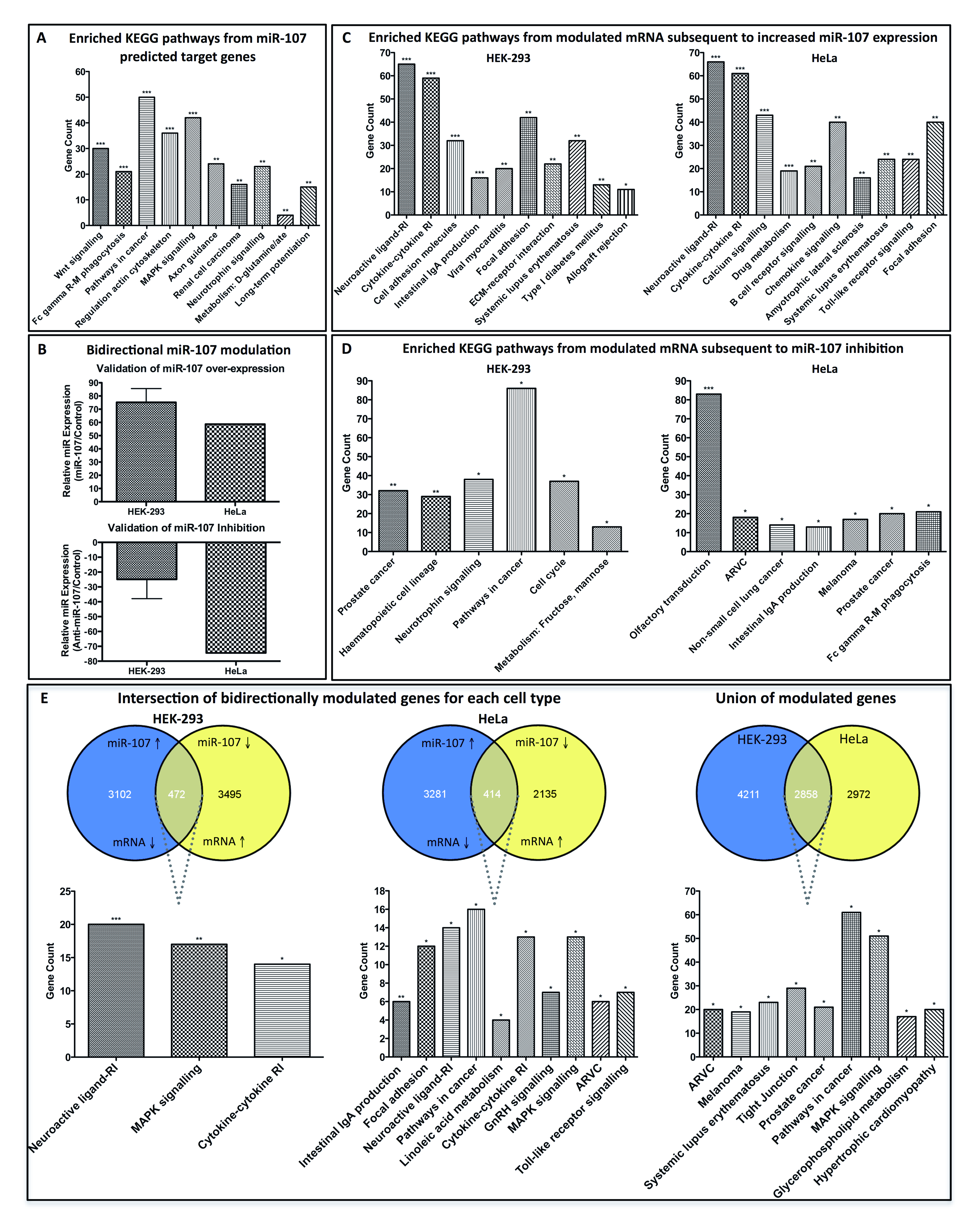

Supplement: Additional file 5 — Figure S4. Bidirectional modulation of miR-107 expression. Panel A shows enriched KEGG pathways from predicted miR-107 target genes. Panel B shows the modulation of miR-107 expression levels in HEK-293 and HeLa cell types, indicative of miR-107 over-expression and inhibition. Panel C illustrates enriched KEGG pathways from modulated mRNA subsequent to miR-107 over-expression in HEK-293 and HeLa cell models. Panel D illustrates enriched KEGG pathways from modulated mRNA subsequent to miR-107 inhibition in HEK-293 and HeLa cell models. Panel E shows Venn diagrams and subsequent KEGG pathways analyses for the intersection of bidirectionally modulated genes in each cell type; and for the union of modulated genes across multiple cell models. The intersection of bidirectionally-modulated genes identifies genes modulated by both miR-107 over-expression and inhibition in each cell type. Genes modulated by either over-expression or inhibition were considered for the union of modulated genes across multiple cell types. The subsequent KEGG pathways analyses on these genes of interest revealed significantly enriched pathways, as evident in the bottom half of this panel. R-M: Receptor-mediated. RI: receptor interaction. ECM: Extracellular matrix. ARVC: Arrhythmogenic right ventricular cardiomyopathy. [file 1471-2164-13-561-S5.tiff]
